# Supplementary figures and images for: Effect of the red uniform on the judgment of position or movement used in Wushu Routine, evaluated by practitioners of the modality
Source: PLoS One. 2024 Mar 21;19(3):e0300893. doi: 10.1371/journal.pone.0300893 (PMC10956778; doi:10.1371/journal.pone.0300893)

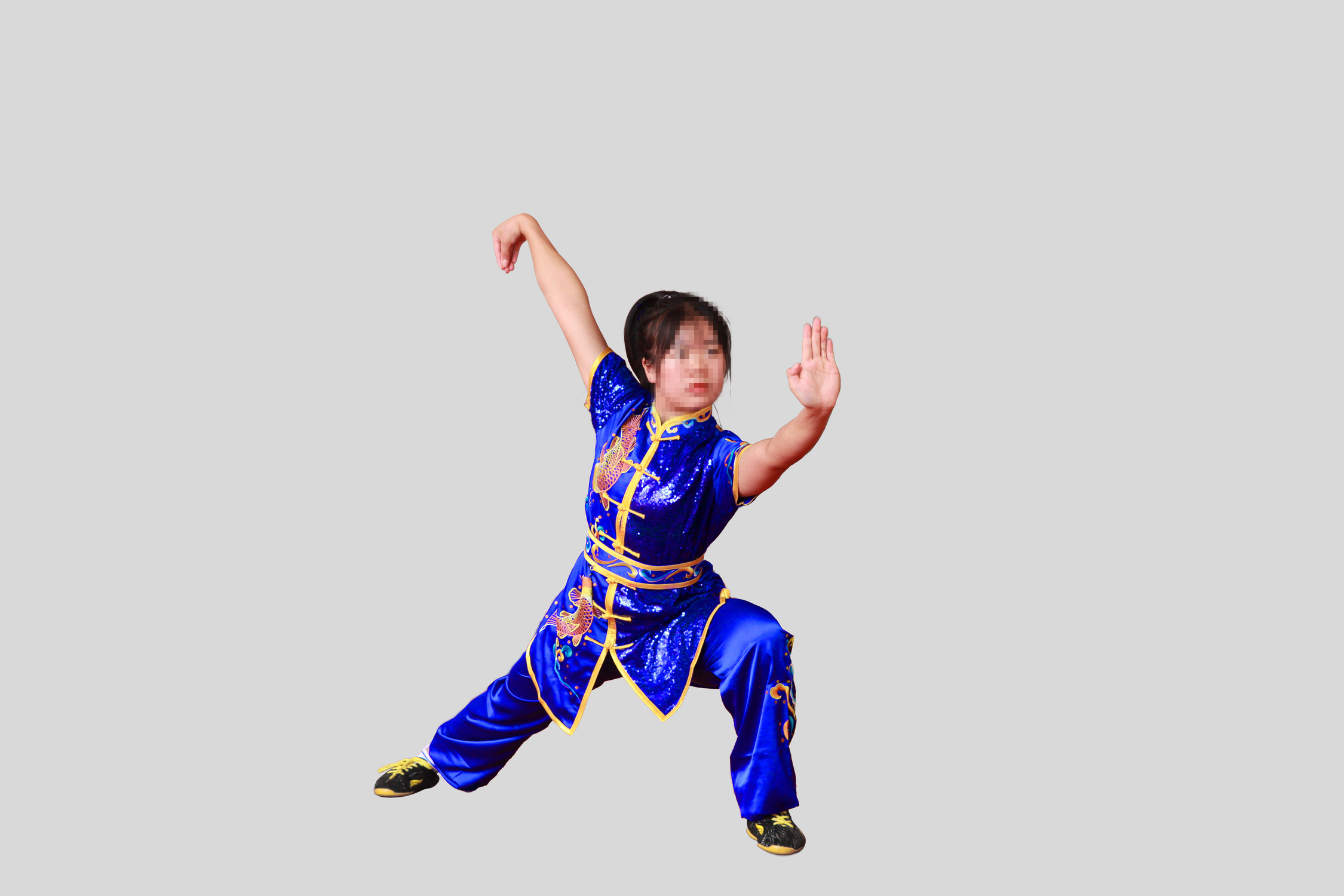

Supplement: S1 File — (ZIP) [file pone.0300893.s001.zip › athlete photos 1/bow-step push palm(female in blue).tif]

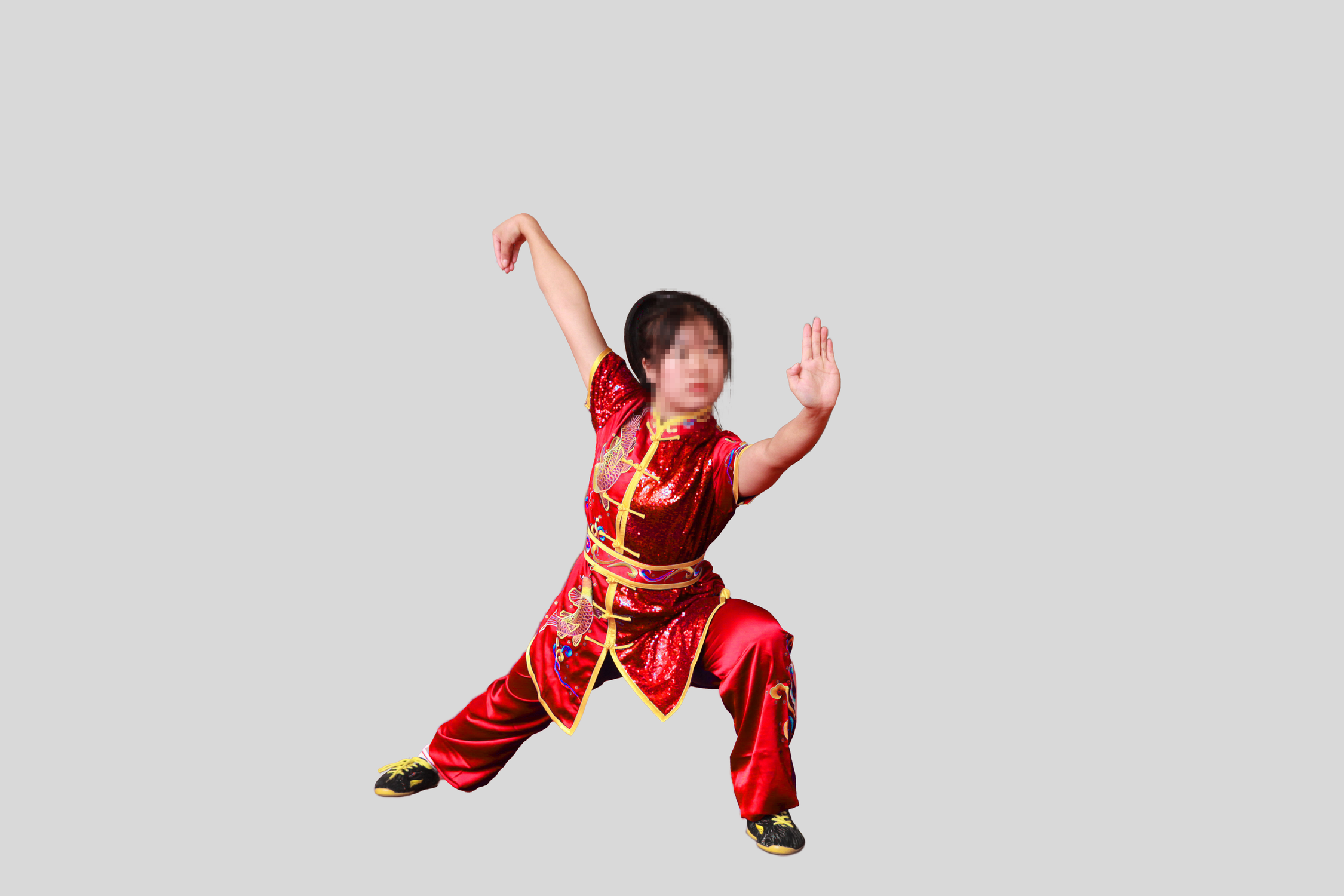

Supplement: S1 File — (ZIP) [file pone.0300893.s001.zip › athlete photos 1/bow-step push palm(female in red).tif]

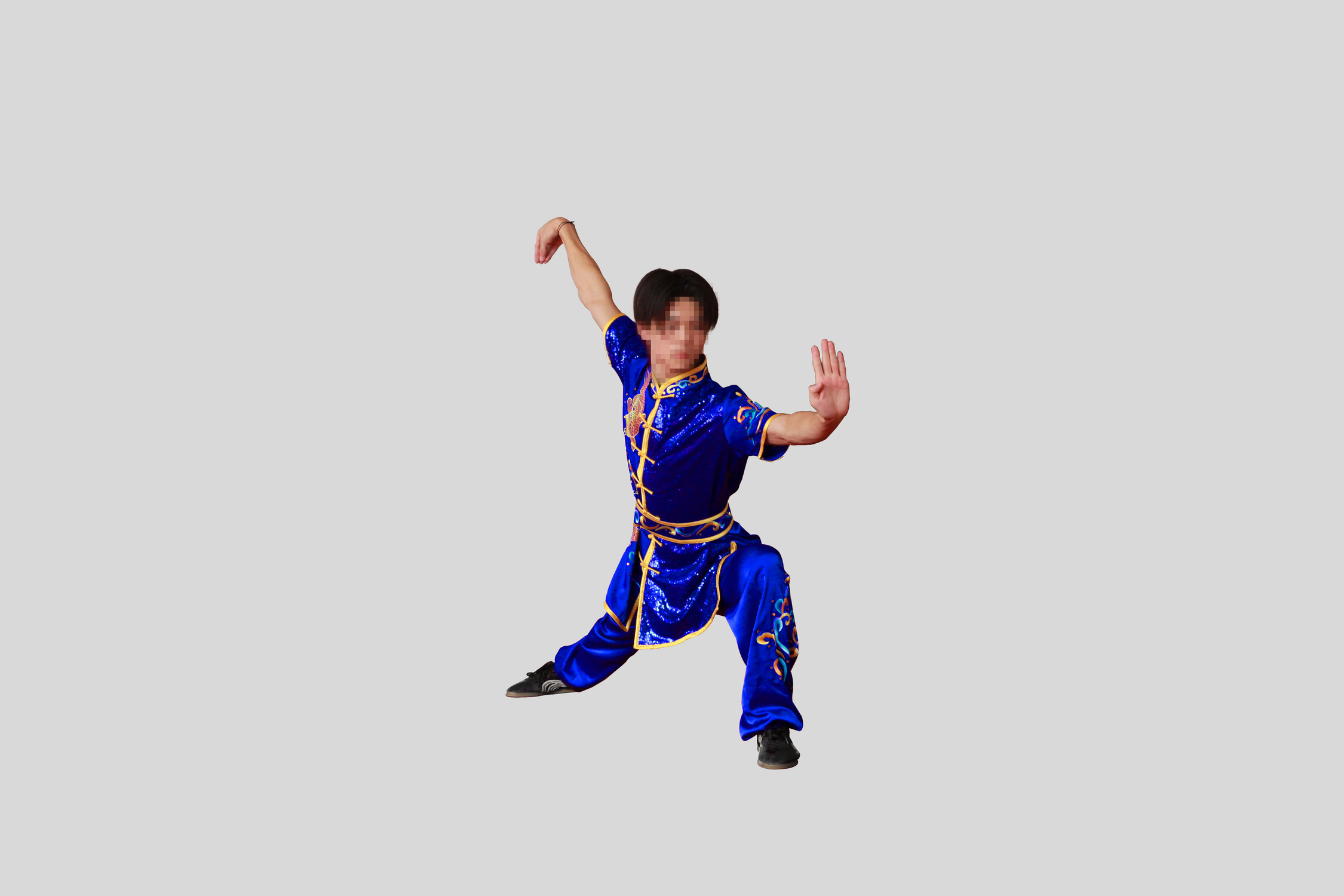

Supplement: S1 File — (ZIP) [file pone.0300893.s001.zip › athlete photos 1/bow-step push palm(male in blue).tif]

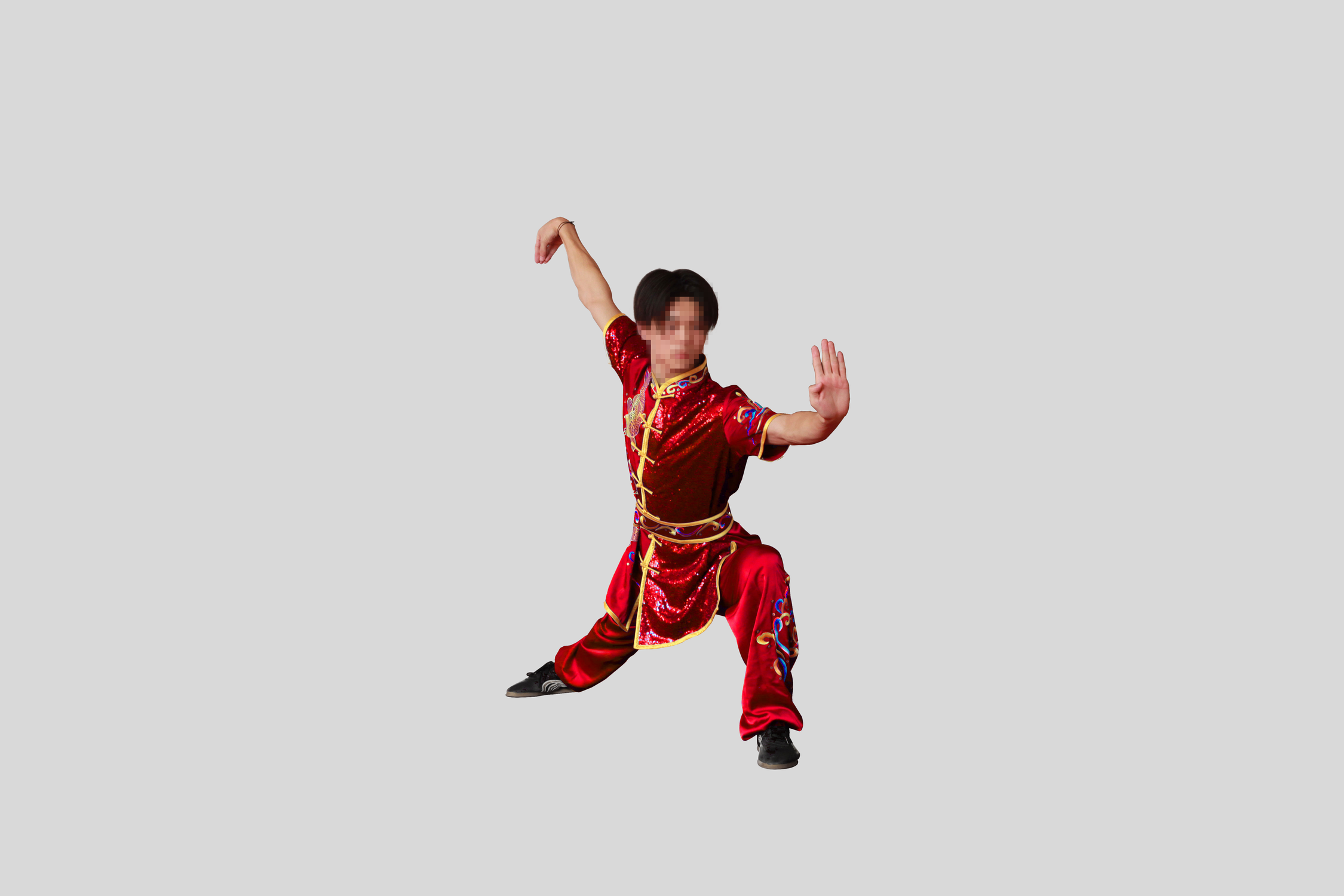

Supplement: S1 File — (ZIP) [file pone.0300893.s001.zip › athlete photos 1/bow-step push palm(male in red).tif]

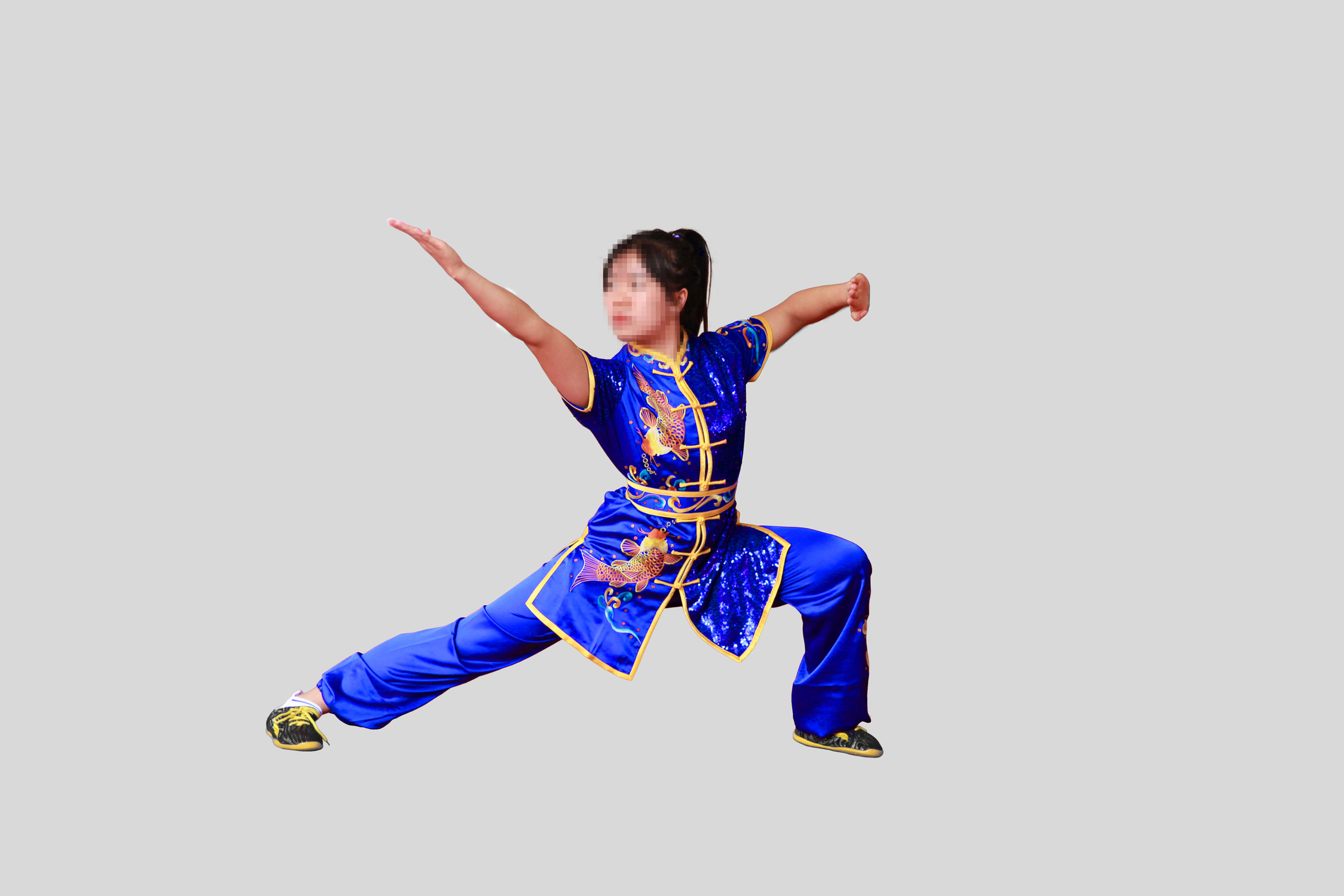

Supplement: S1 File — (ZIP) [file pone.0300893.s001.zip › athlete photos 1/bow-step through palm( female in blue).tif]

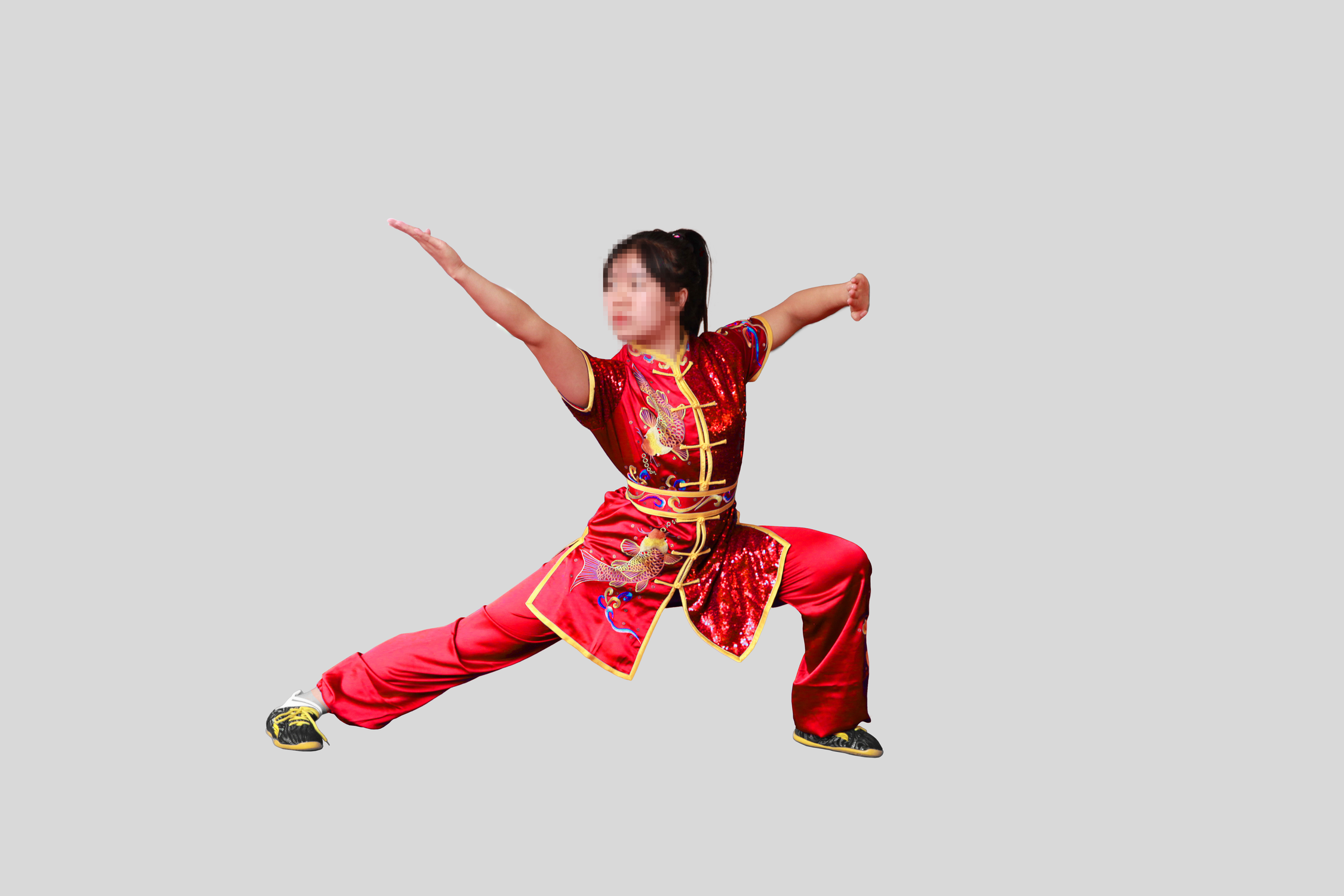

Supplement: S1 File — (ZIP) [file pone.0300893.s001.zip › athlete photos 1/bow-step through palm( female in red).tif]

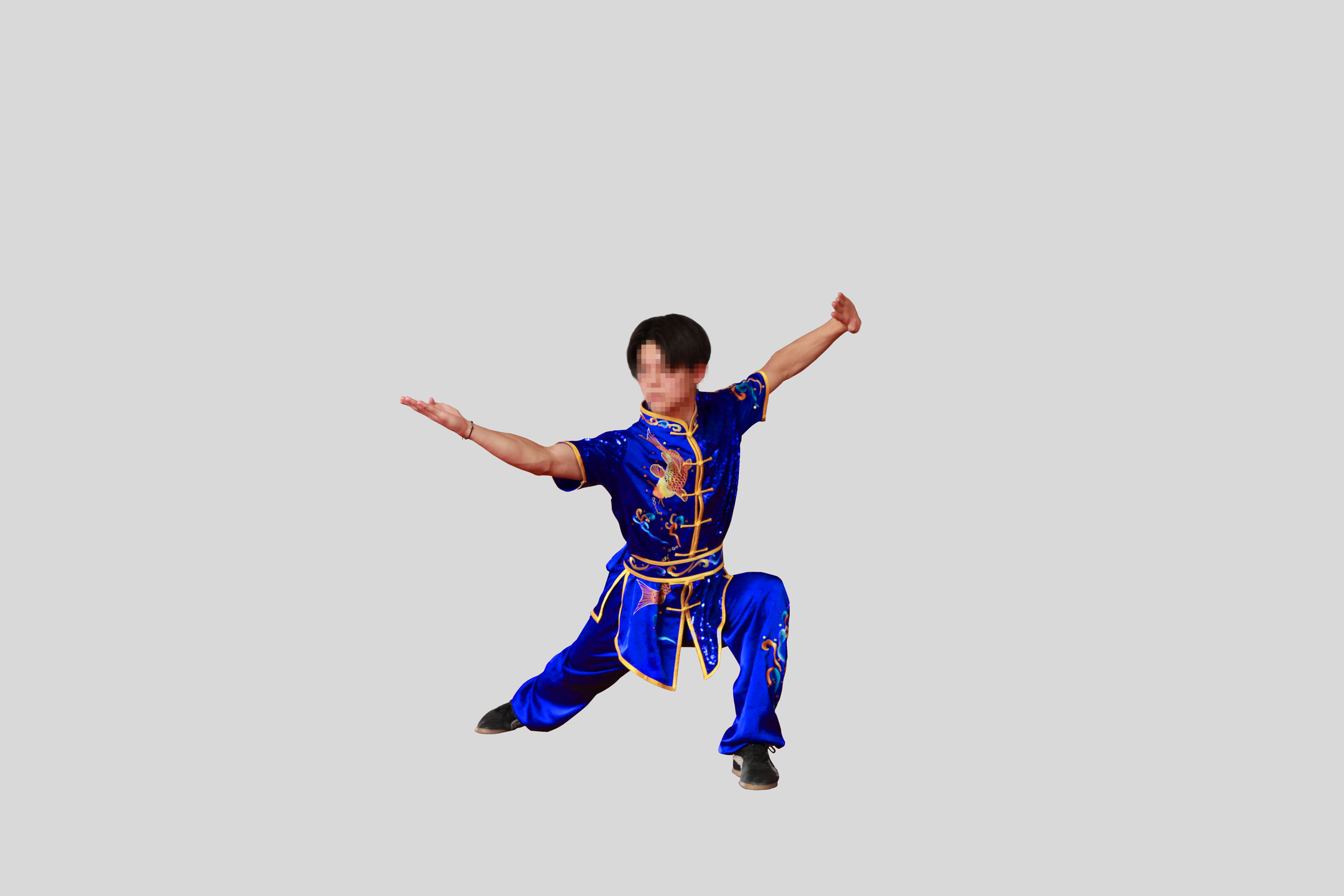

Supplement: S1 File — (ZIP) [file pone.0300893.s001.zip › athlete photos 1/bow-step through palm(male in blue).tif]

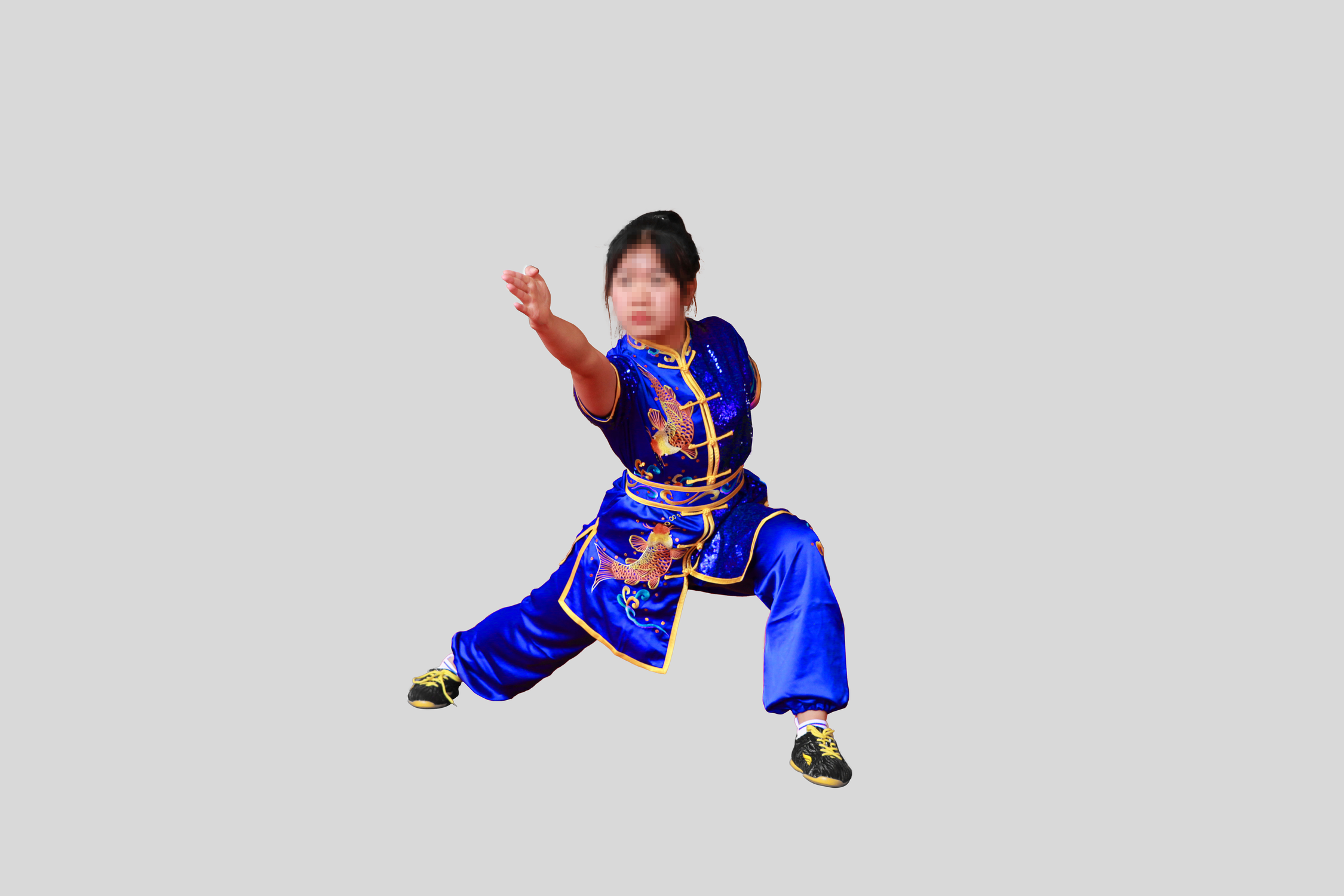

Supplement: S1 File — (ZIP) [file pone.0300893.s001.zip › athlete photos 1/bow-step through palm2( female in blue).tif]

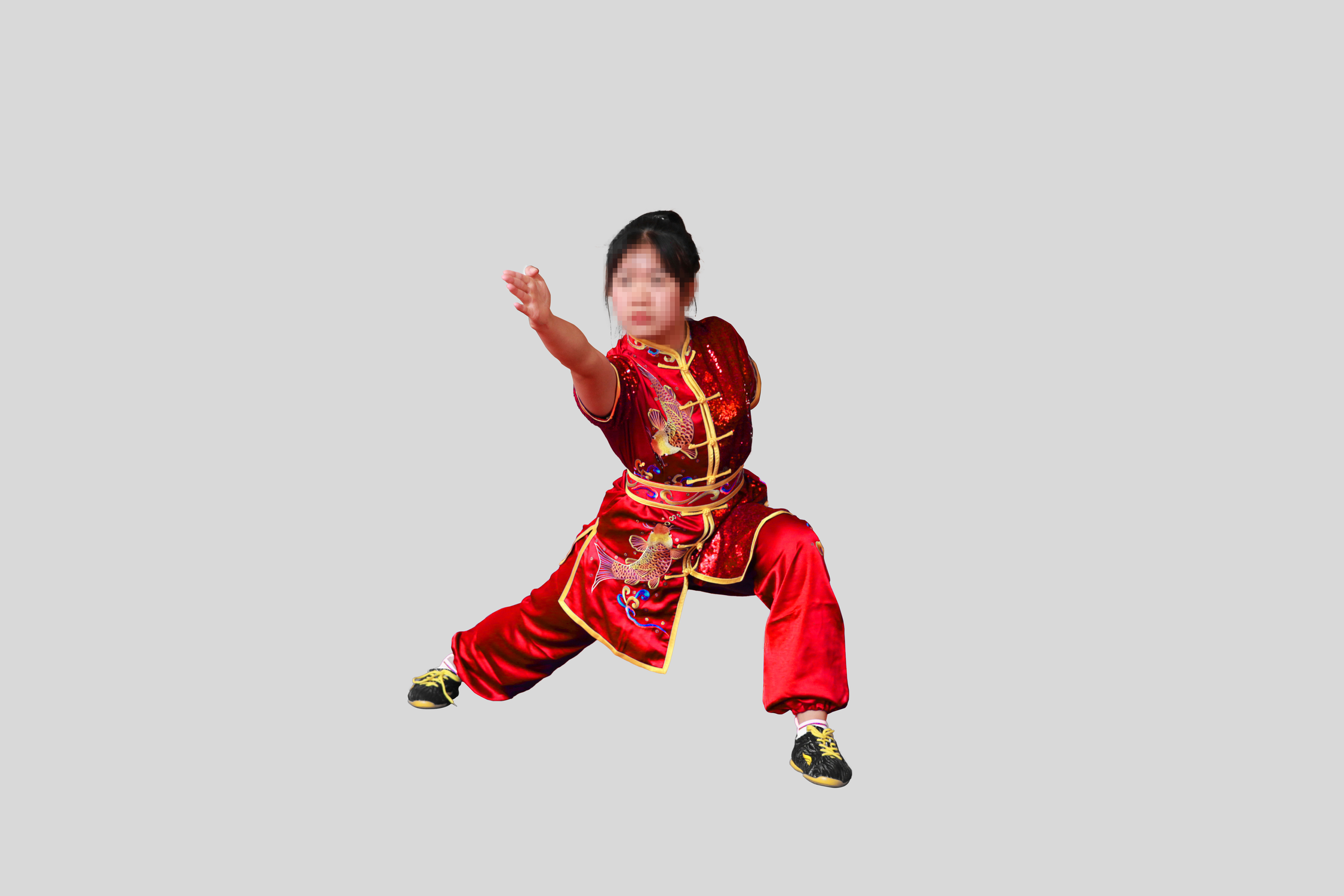

Supplement: S1 File — (ZIP) [file pone.0300893.s001.zip › athlete photos 1/bow-step through palm2( female in red).tif]

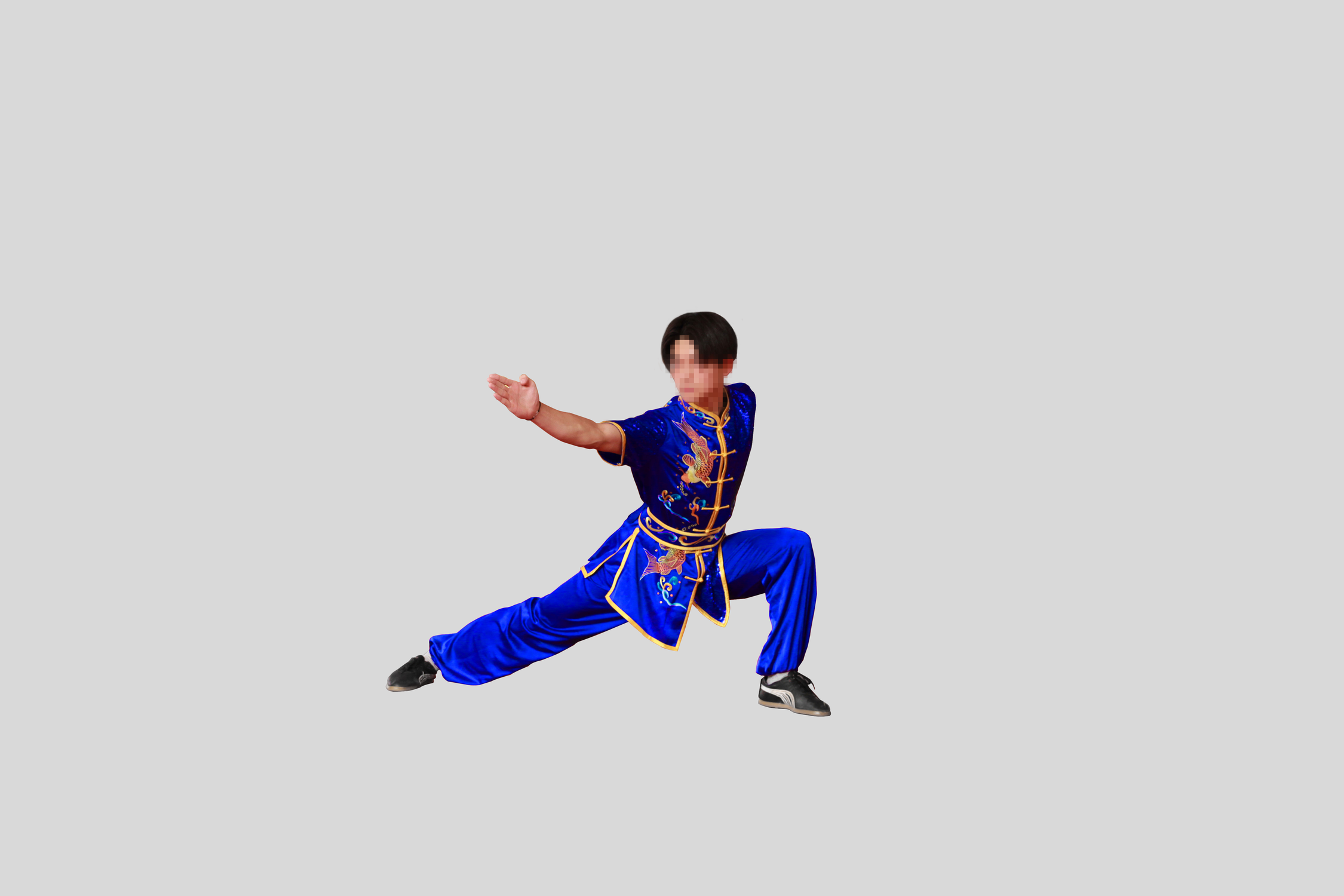

Supplement: S1 File — (ZIP) [file pone.0300893.s001.zip › athlete photos 1/bow-step through palm2(male in blue).tif]

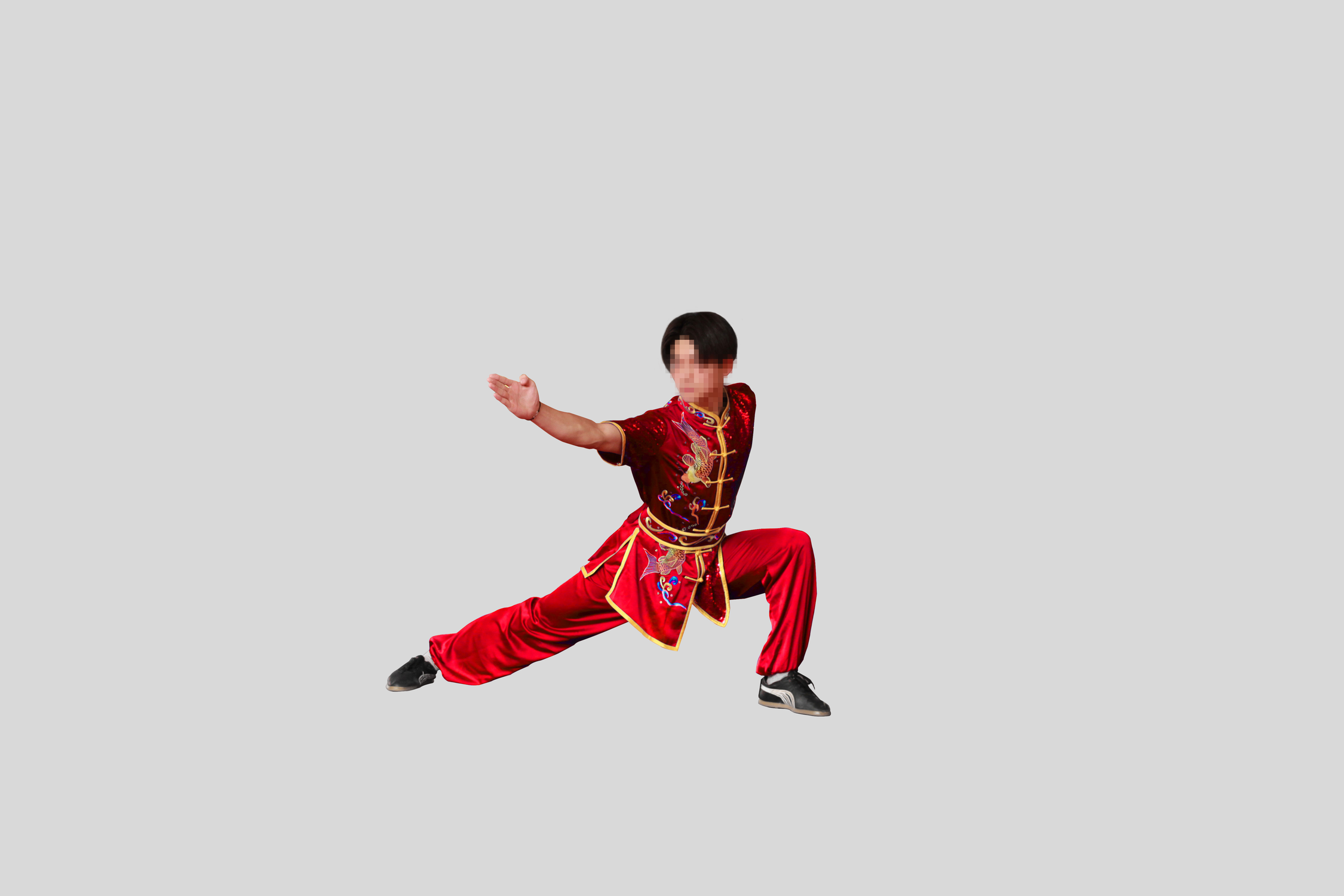

Supplement: S1 File — (ZIP) [file pone.0300893.s001.zip › athlete photos 1/bow-step through palm2(male in red).tif]

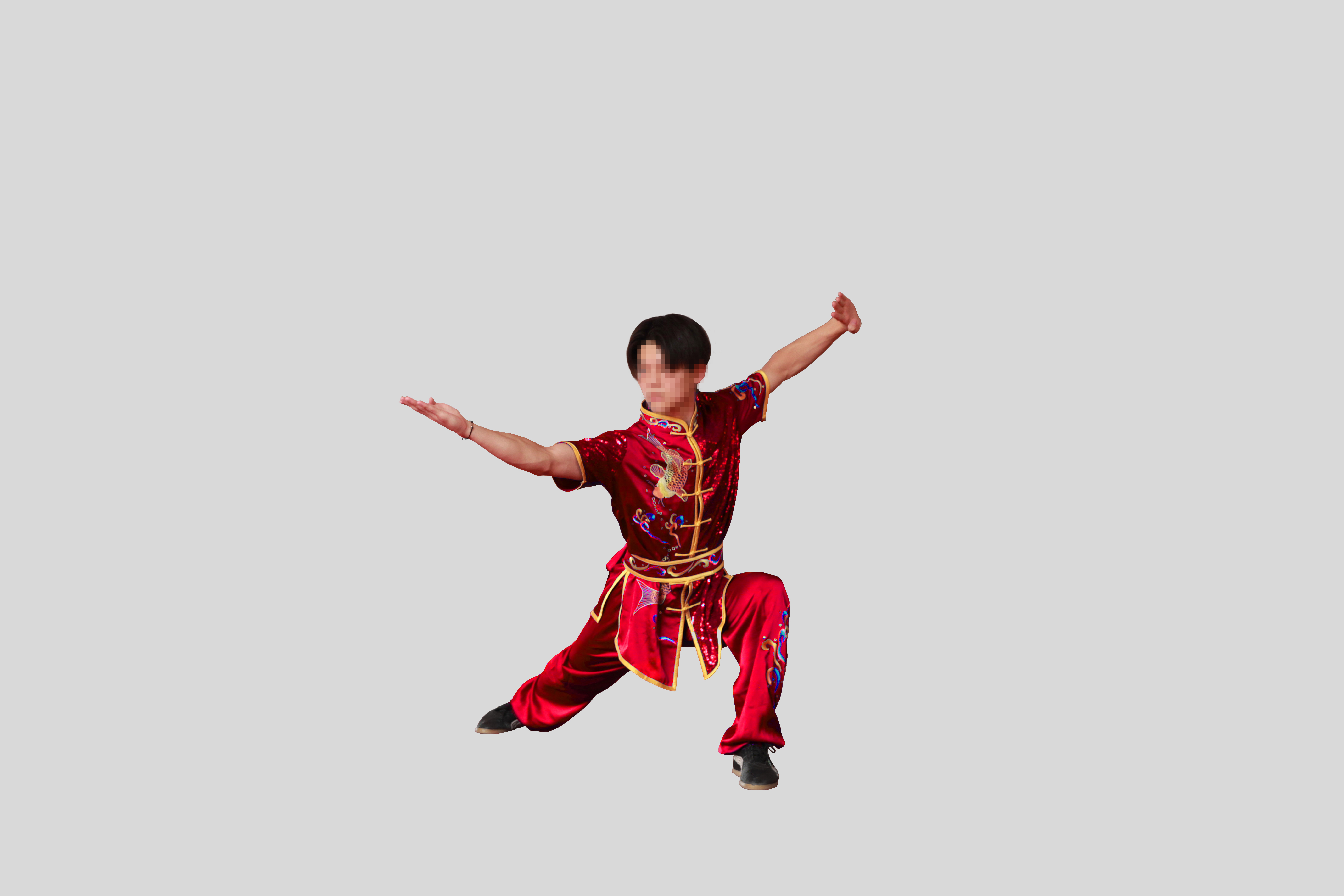

Supplement: S1 File — (ZIP) [file pone.0300893.s001.zip › athlete photos 1/bow-step through palm( male in red).jpg]

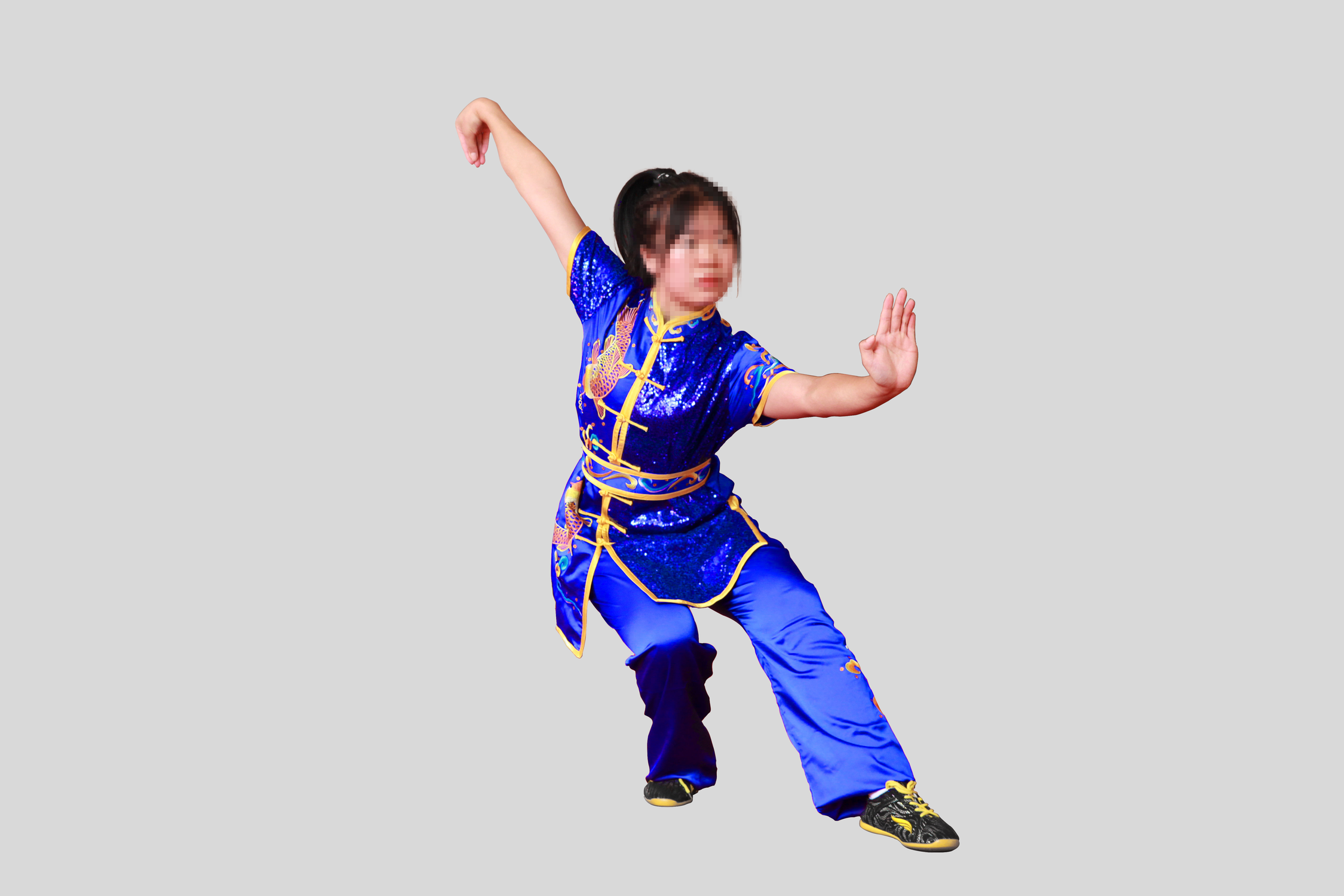

Supplement: S2 File — (ZIP) [file pone.0300893.s002.zip › athlete photos 2/empty-step push palm(female in bule).tif]

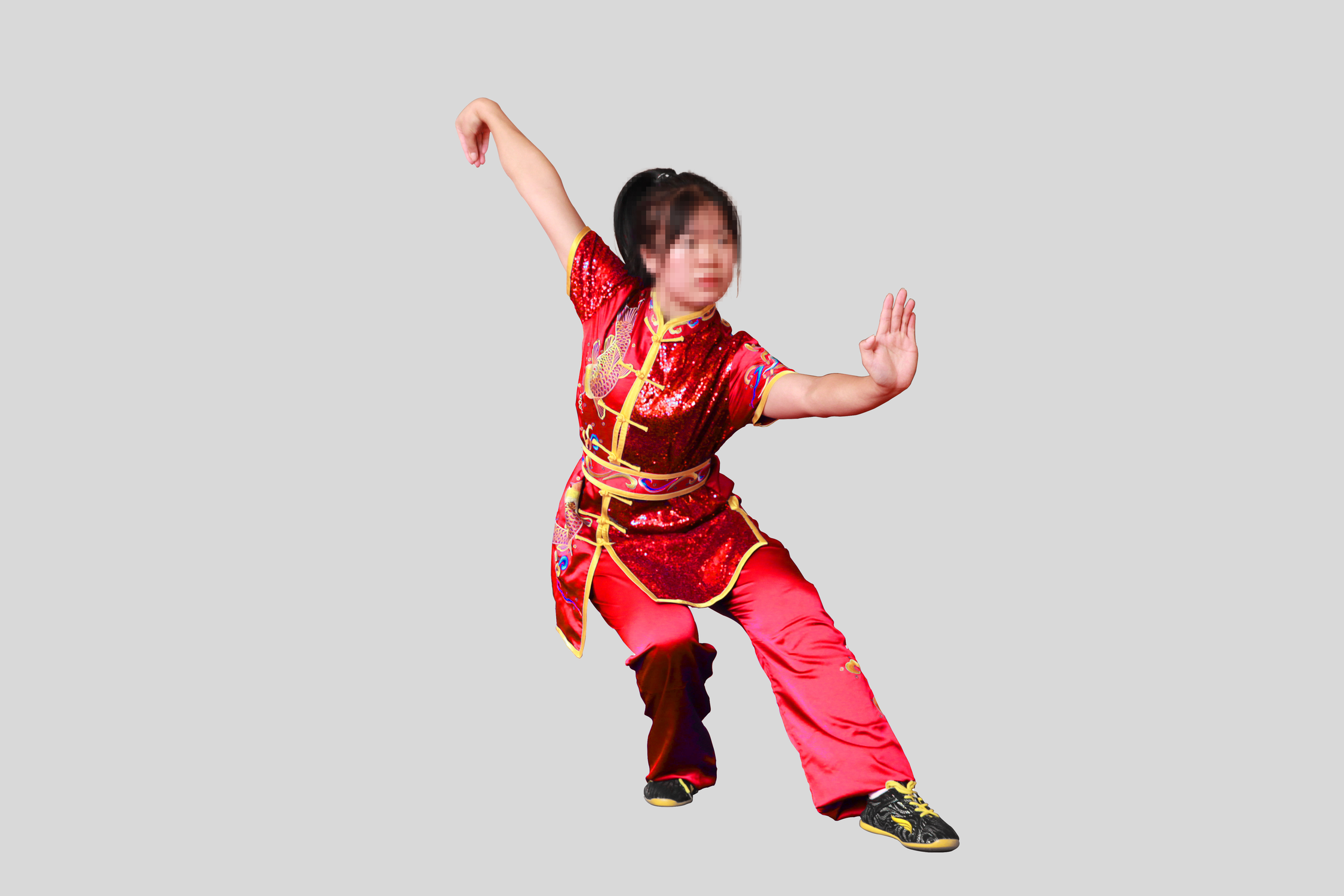

Supplement: S2 File — (ZIP) [file pone.0300893.s002.zip › athlete photos 2/empty-step push palm(female in red).tif]

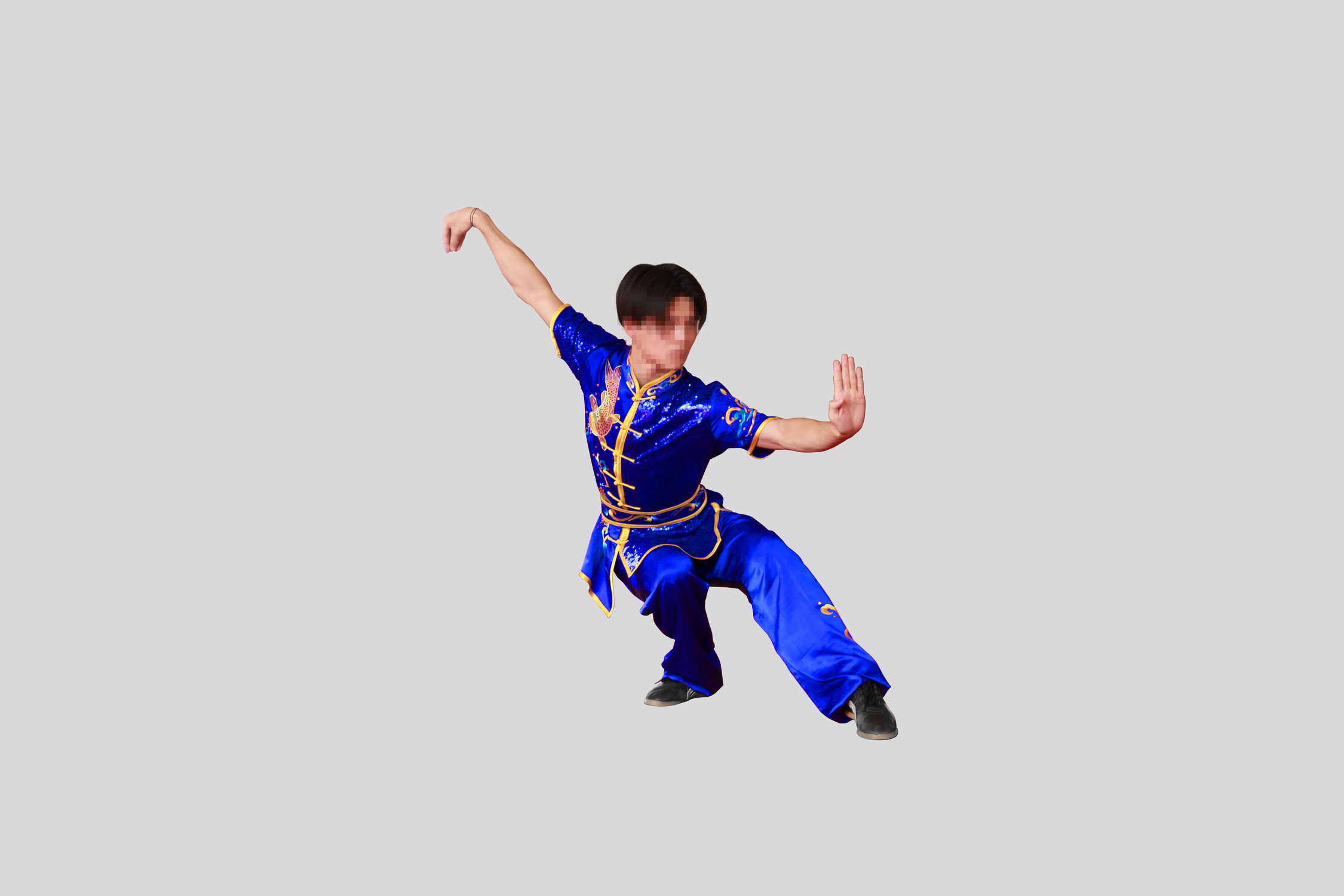

Supplement: S2 File — (ZIP) [file pone.0300893.s002.zip › athlete photos 2/empty-step push palm(male in bule).tif]

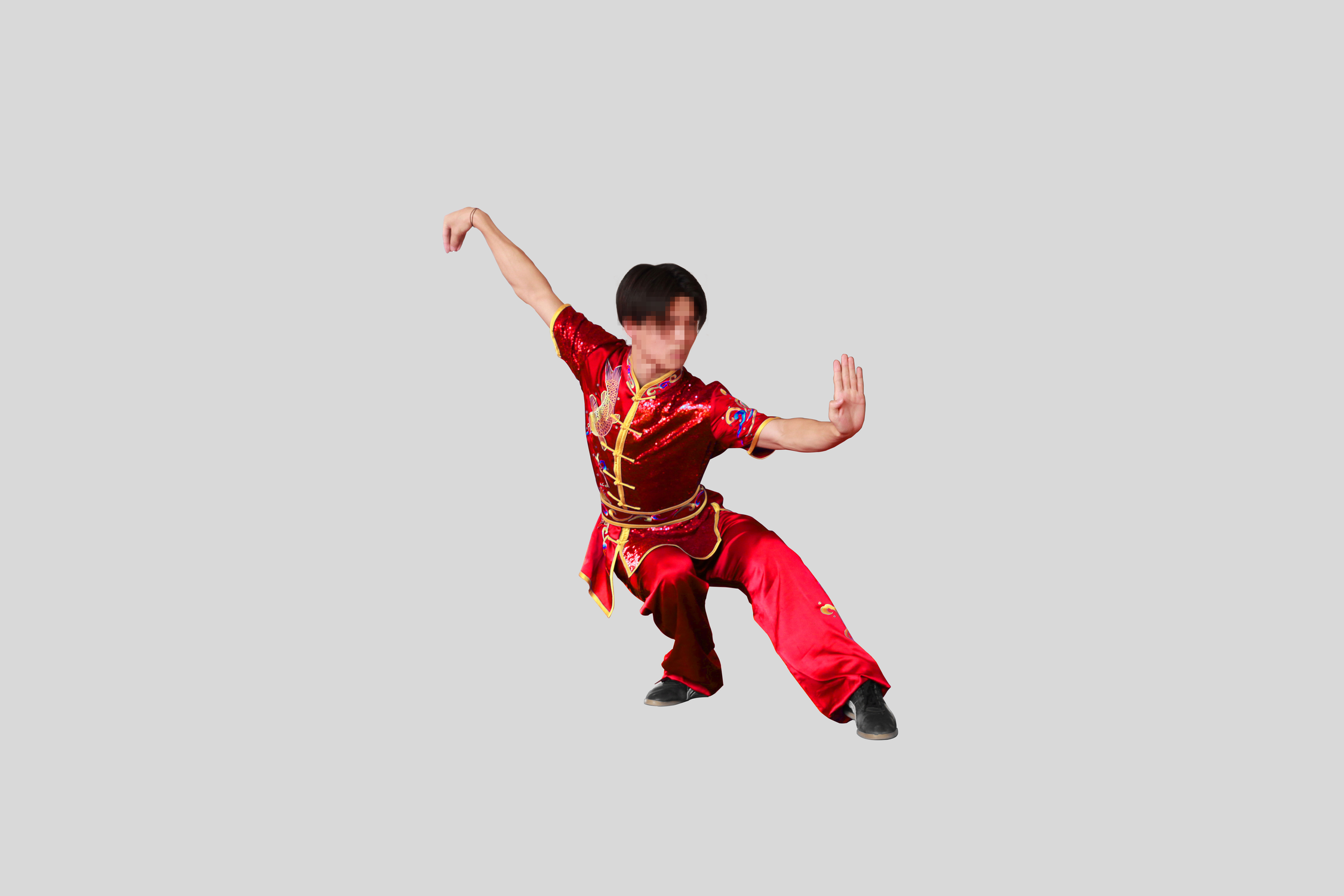

Supplement: S2 File — (ZIP) [file pone.0300893.s002.zip › athlete photos 2/empty-step push palm(male in red).tif]

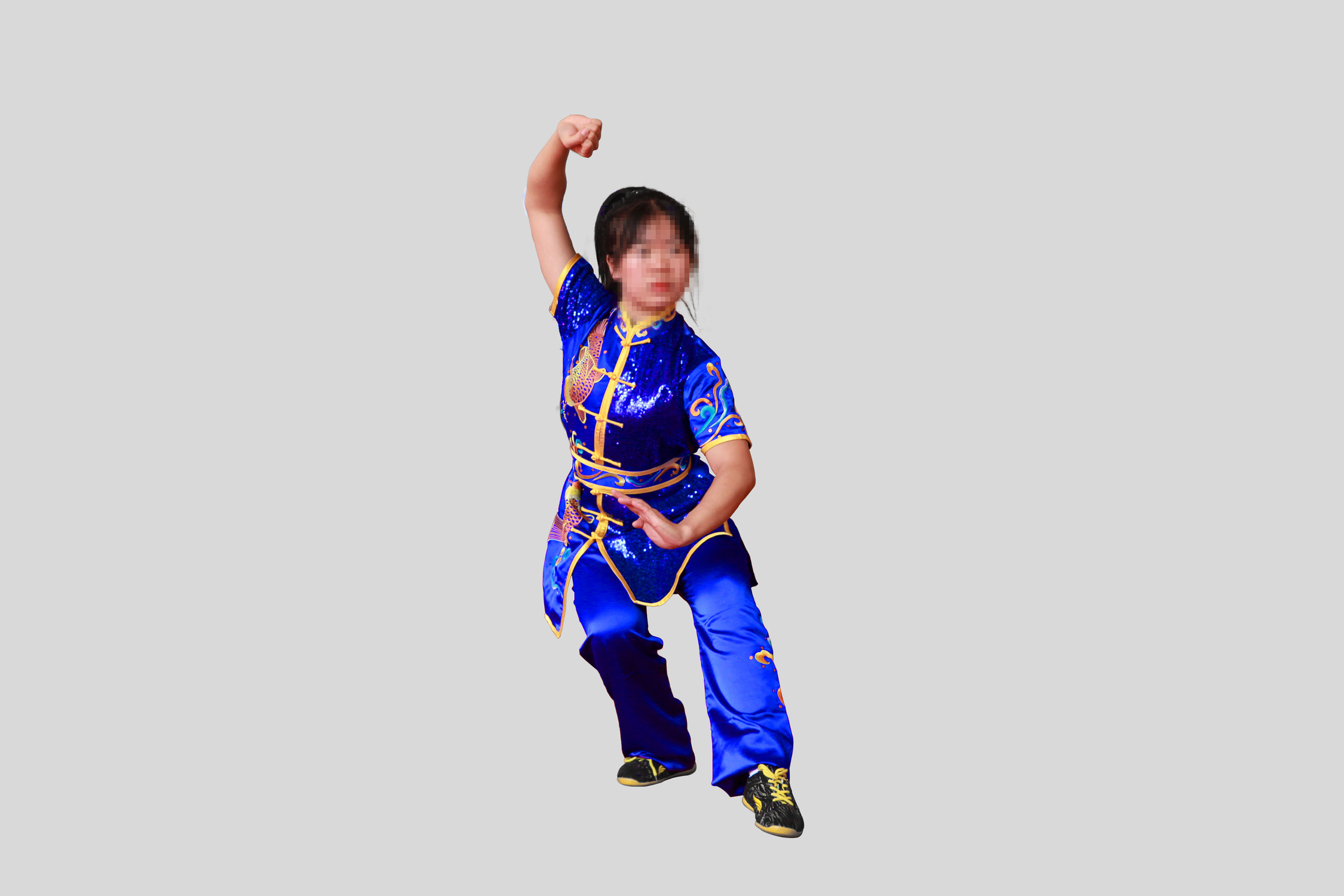

Supplement: S2 File — (ZIP) [file pone.0300893.s002.zip › athlete photos 2/empty-step show palm (female in blue).tif]

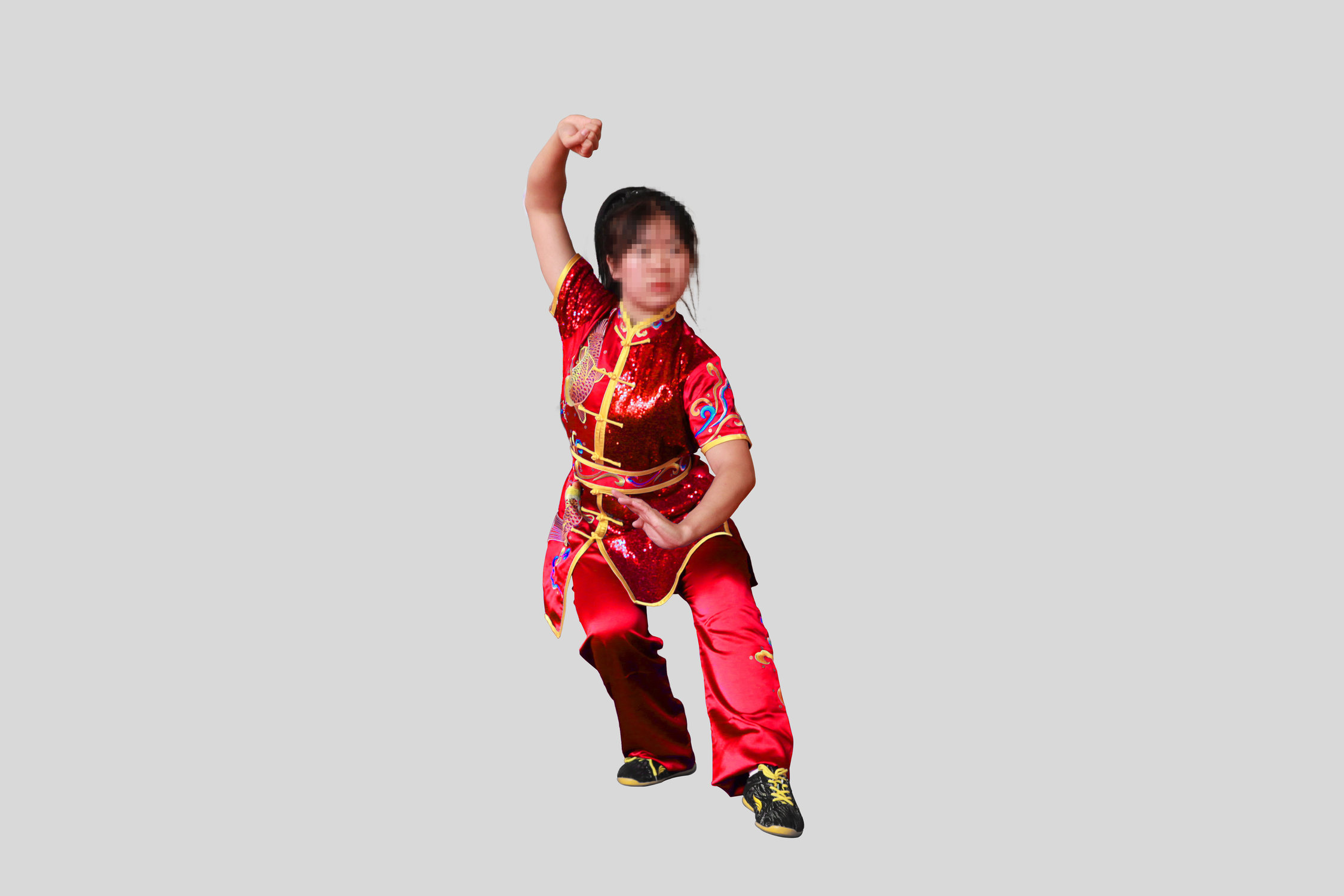

Supplement: S2 File — (ZIP) [file pone.0300893.s002.zip › athlete photos 2/empty-step show palm (female in red).tif]

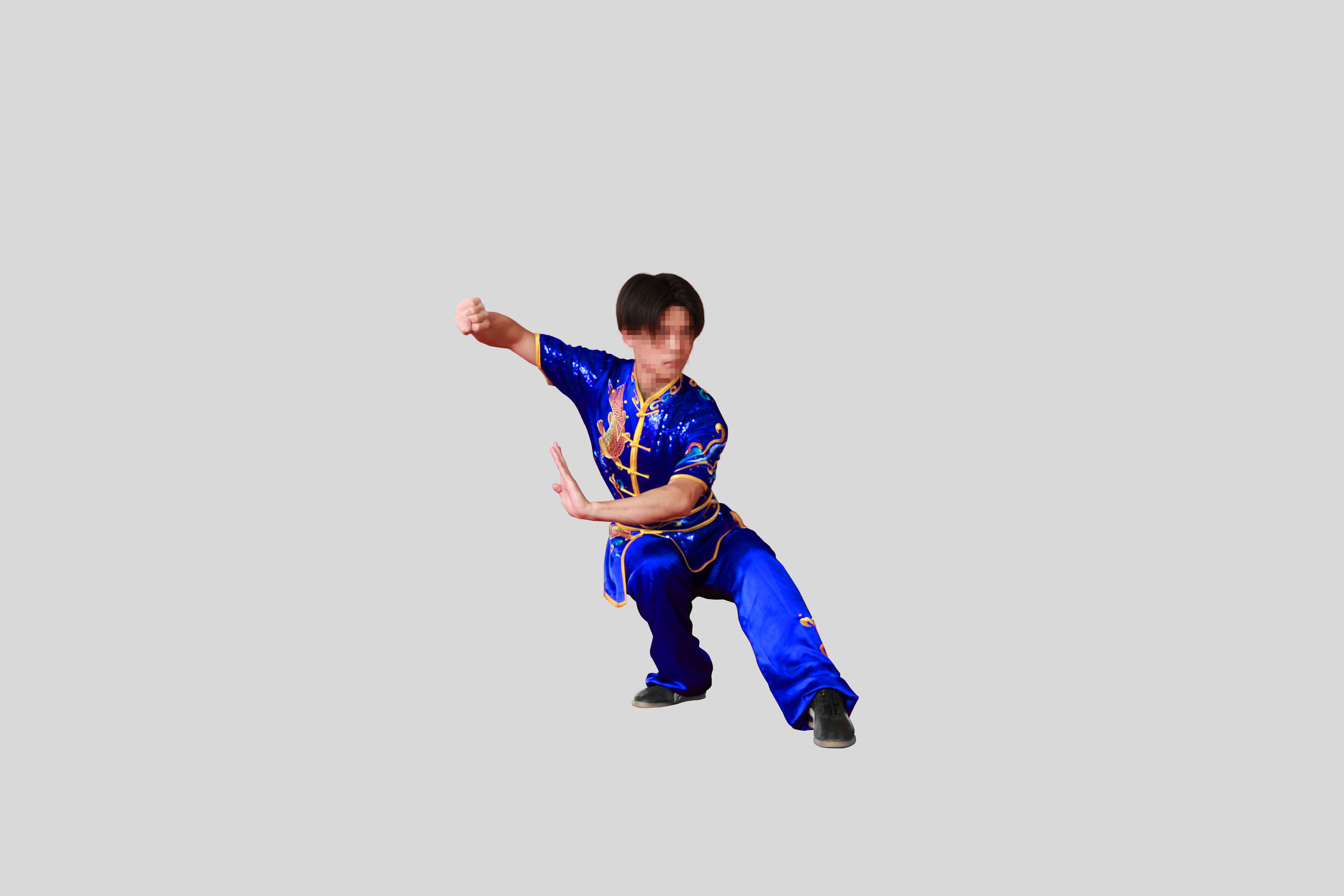

Supplement: S2 File — (ZIP) [file pone.0300893.s002.zip › athlete photos 2/empty-step show palm (male in blue).tif]

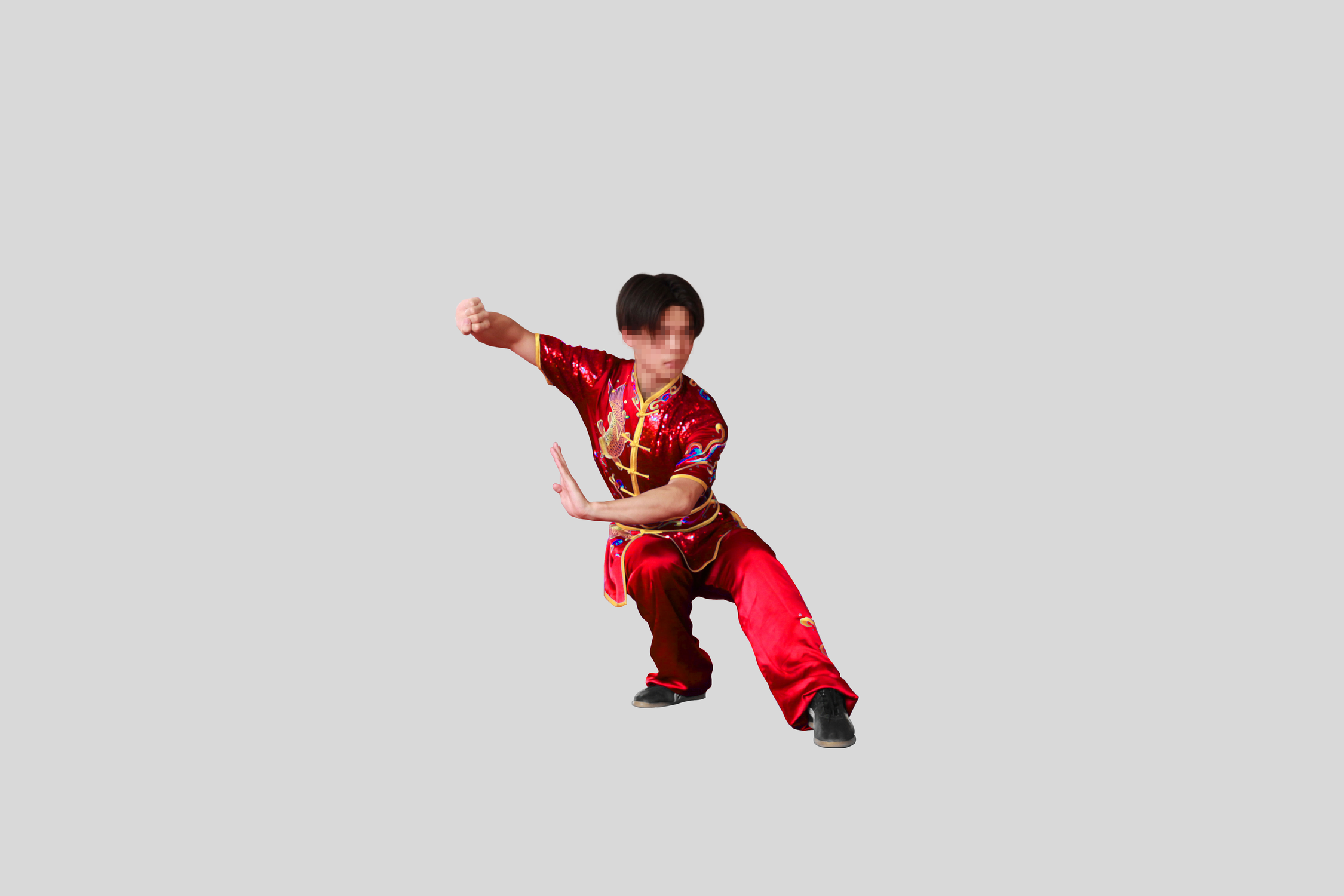

Supplement: S2 File — (ZIP) [file pone.0300893.s002.zip › athlete photos 2/empty-step show palm (male in red).tif]

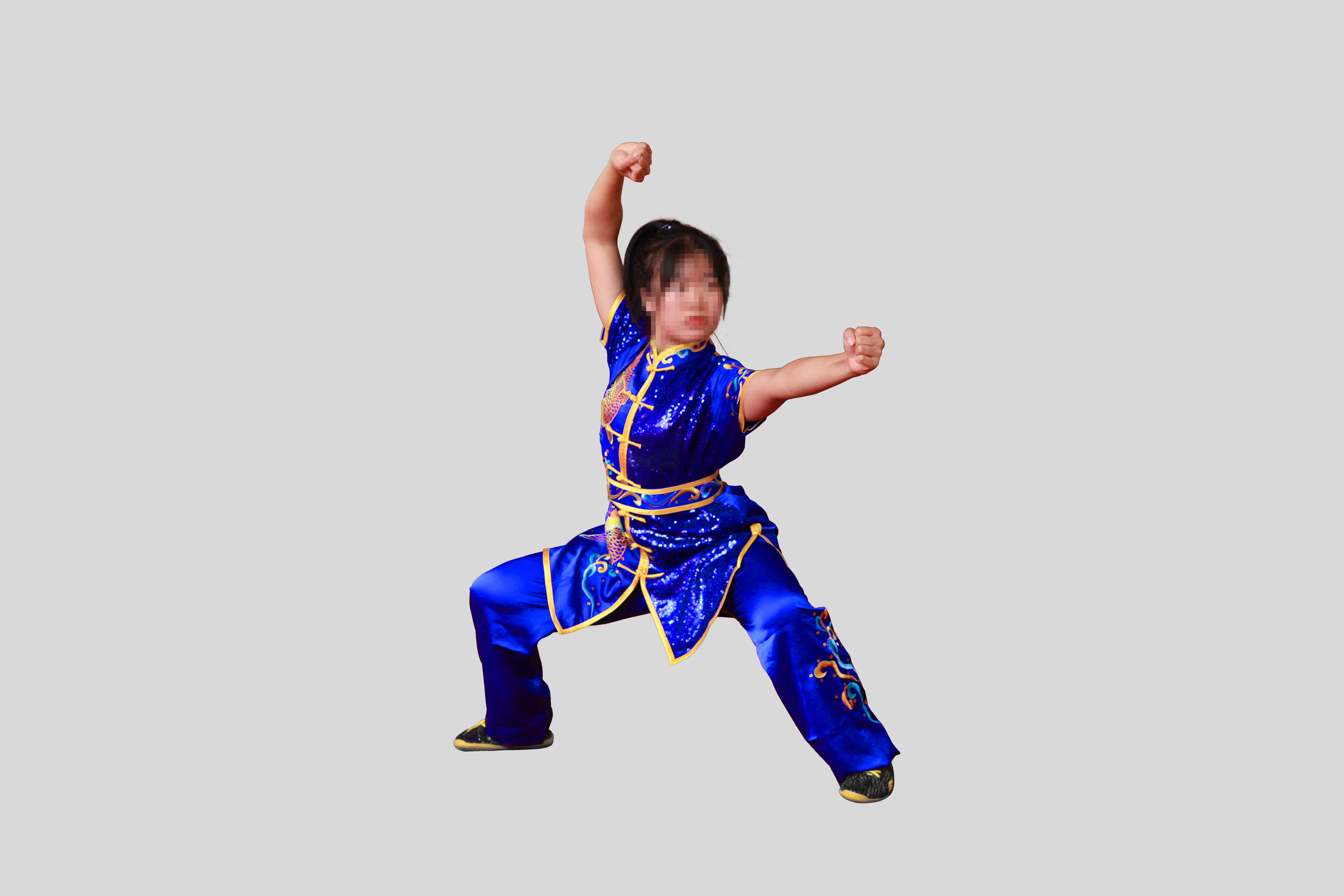

Supplement: S2 File — (ZIP) [file pone.0300893.s002.zip › athlete photos 2/horse-step strike fist(female in blue).tif]

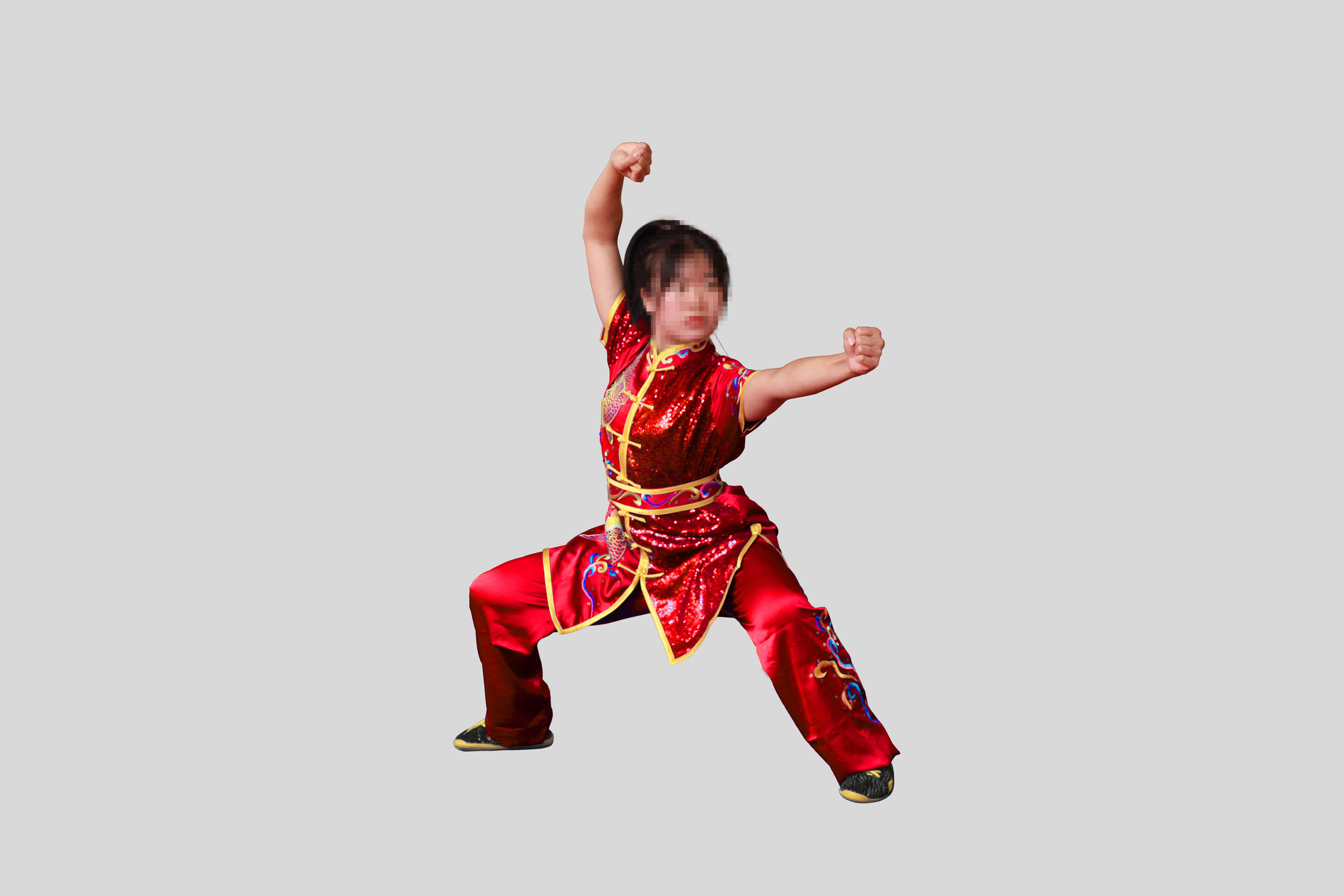

Supplement: S2 File — (ZIP) [file pone.0300893.s002.zip › athlete photos 2/horse-step strike fist(female in red).tif]

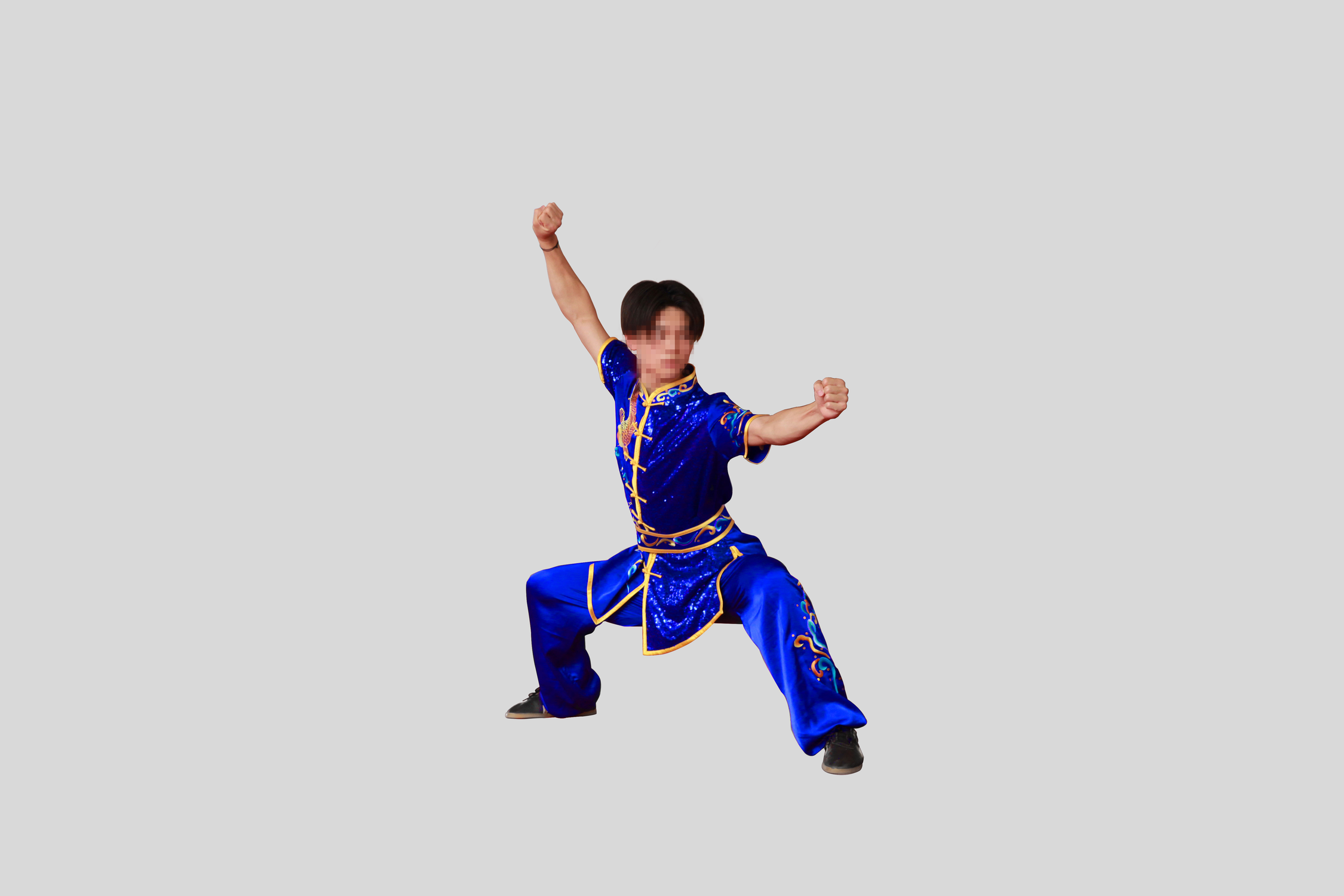

Supplement: S2 File — (ZIP) [file pone.0300893.s002.zip › athlete photos 2/horse-step strike fist(male in blue).tif]

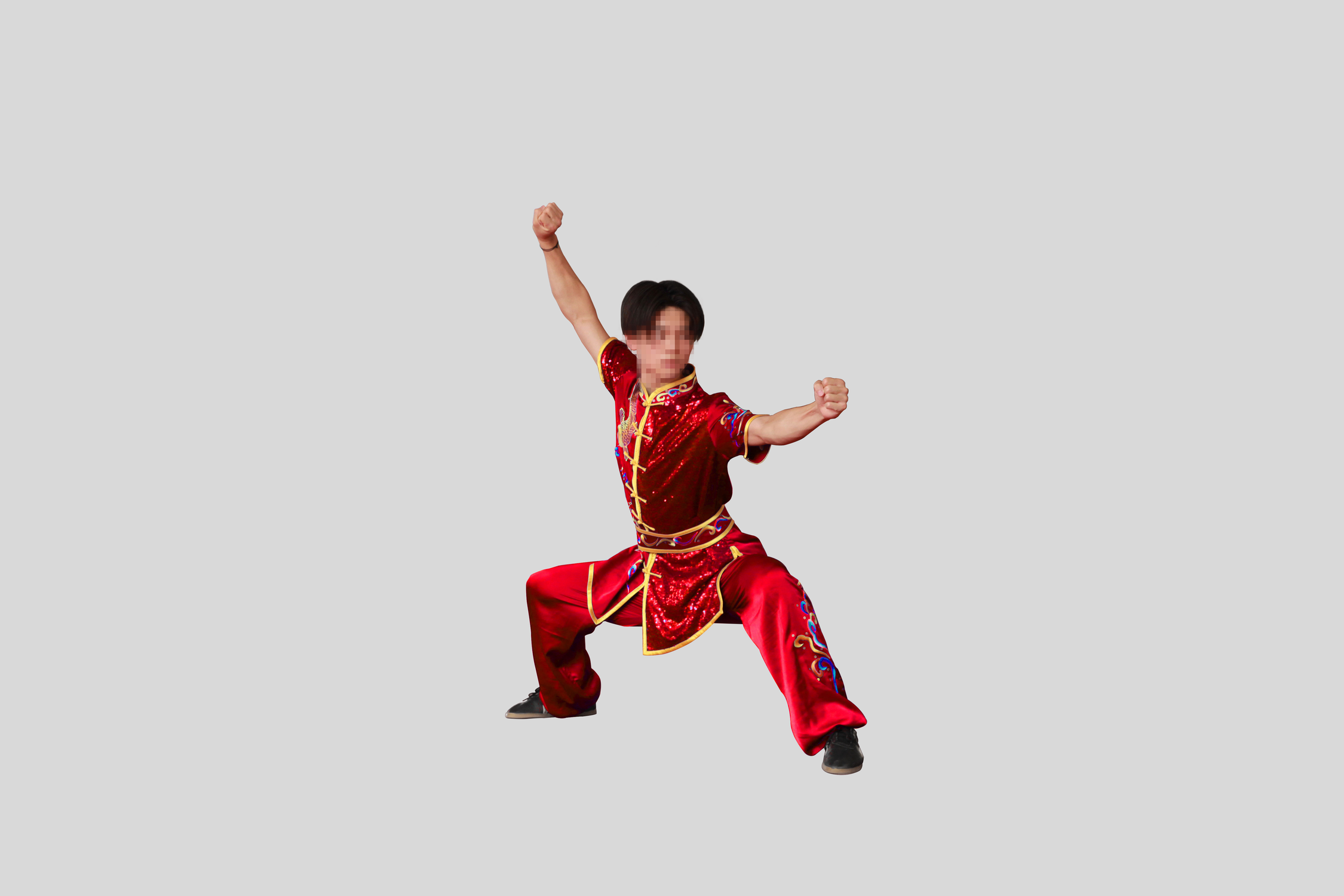

Supplement: S2 File — (ZIP) [file pone.0300893.s002.zip › athlete photos 2/horse-step strike fist(male in red).tif]

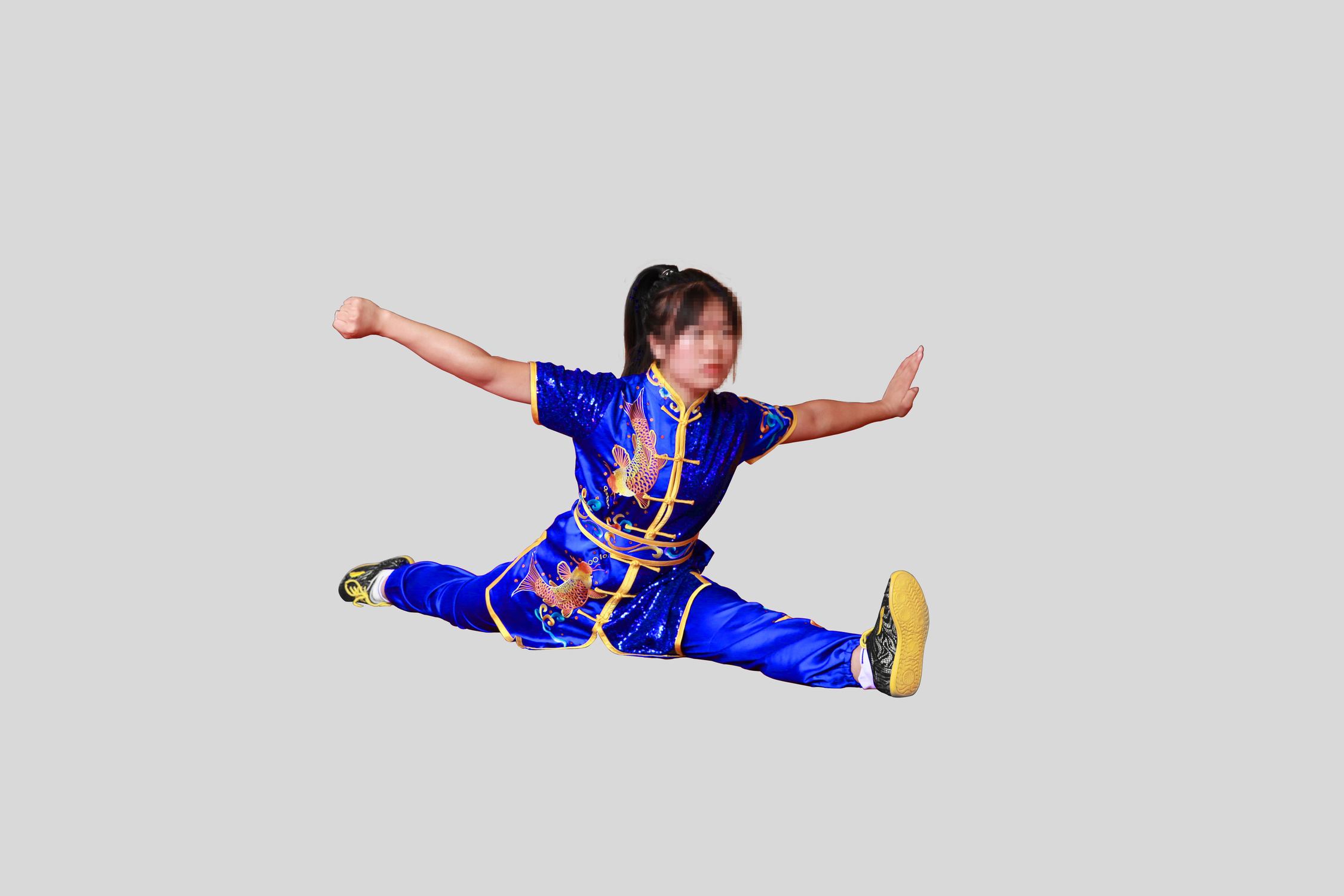

Supplement: S2 File — (ZIP) [file pone.0300893.s002.zip › athlete photos 2/longitudinal split(female in blue).tif]

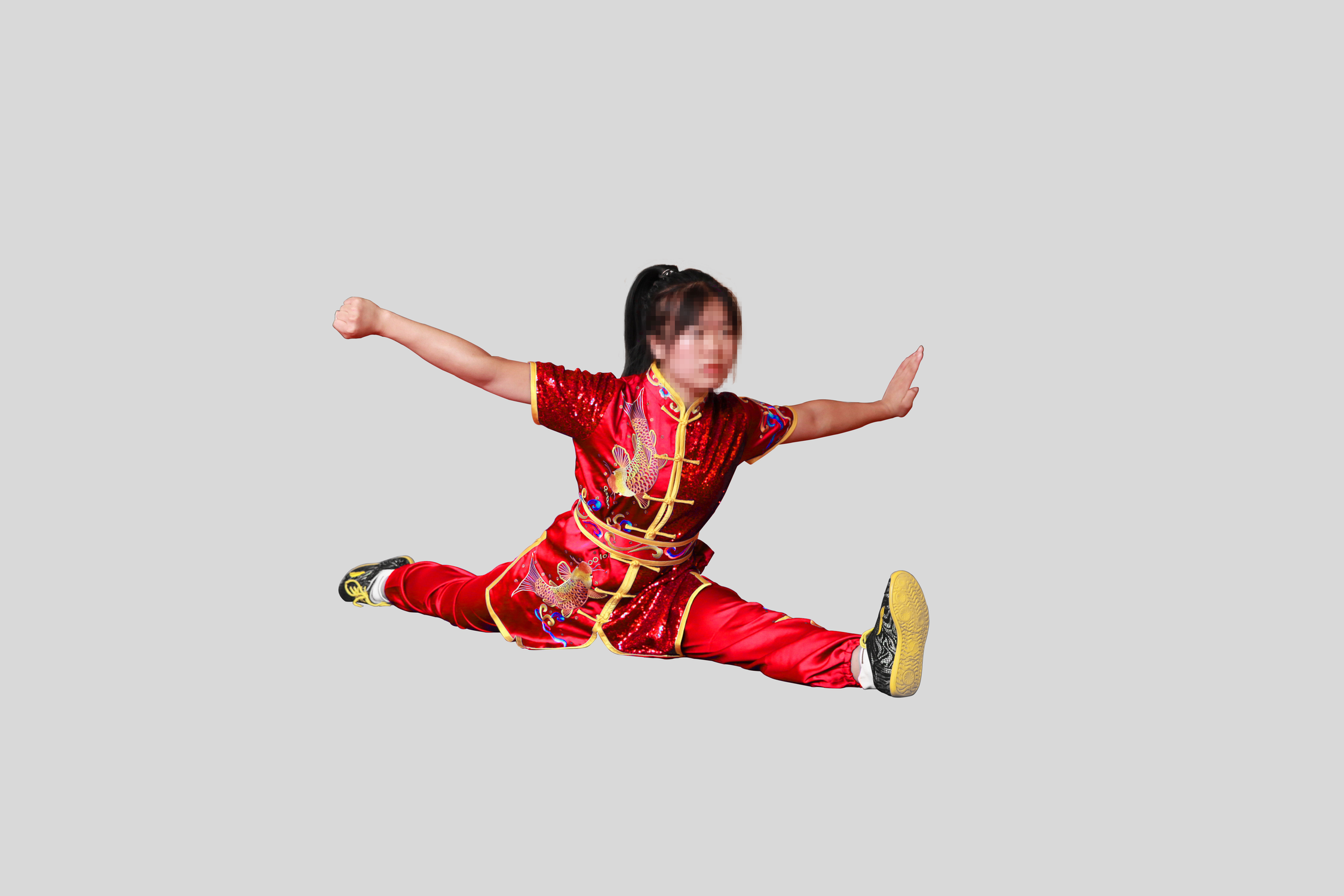

Supplement: S2 File — (ZIP) [file pone.0300893.s002.zip › athlete photos 2/longitudinal split(female in red).tif]

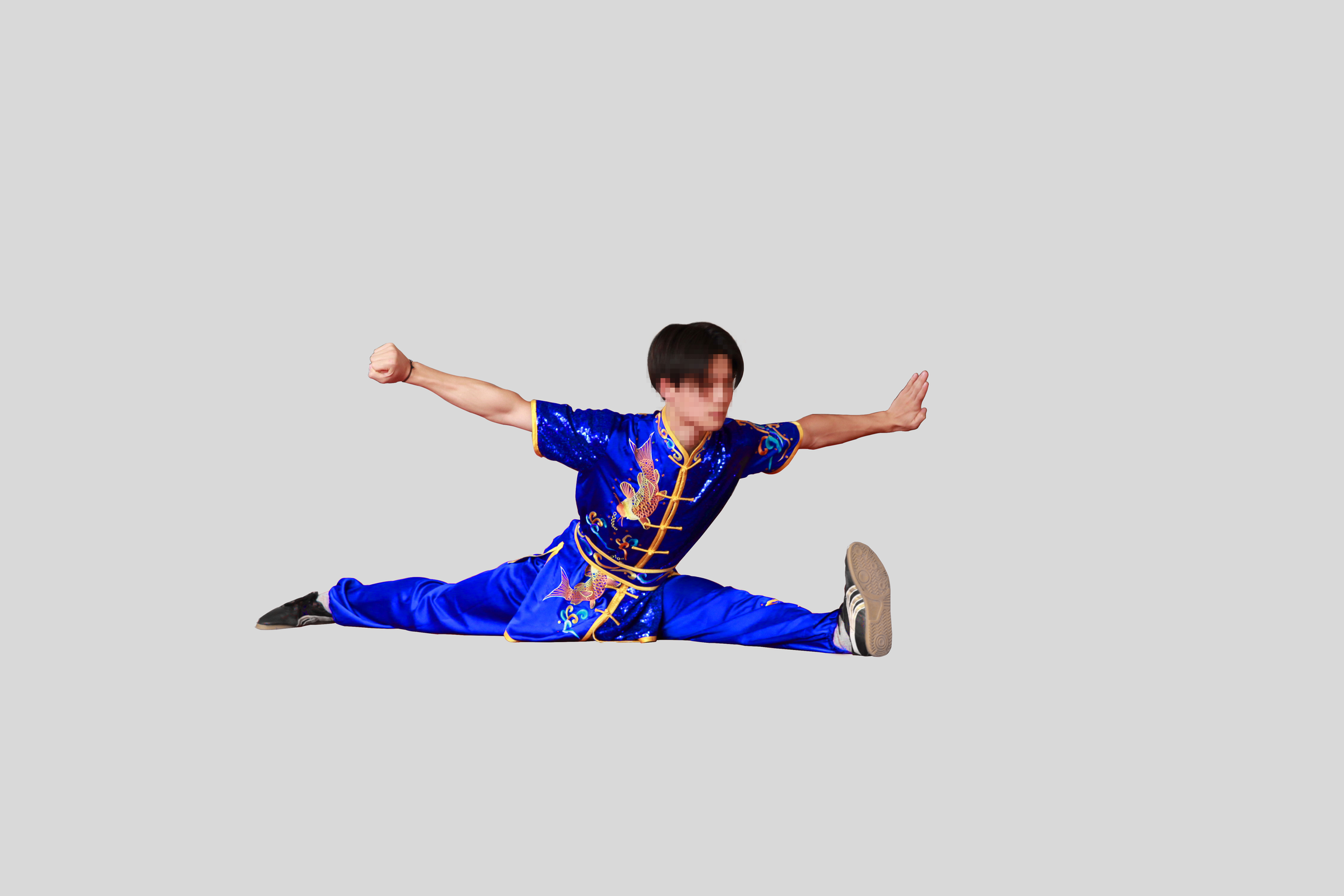

Supplement: S3 File — (ZIP) [file pone.0300893.s003.zip › athlete photos 3/longitudinal split(male in blue).tif]

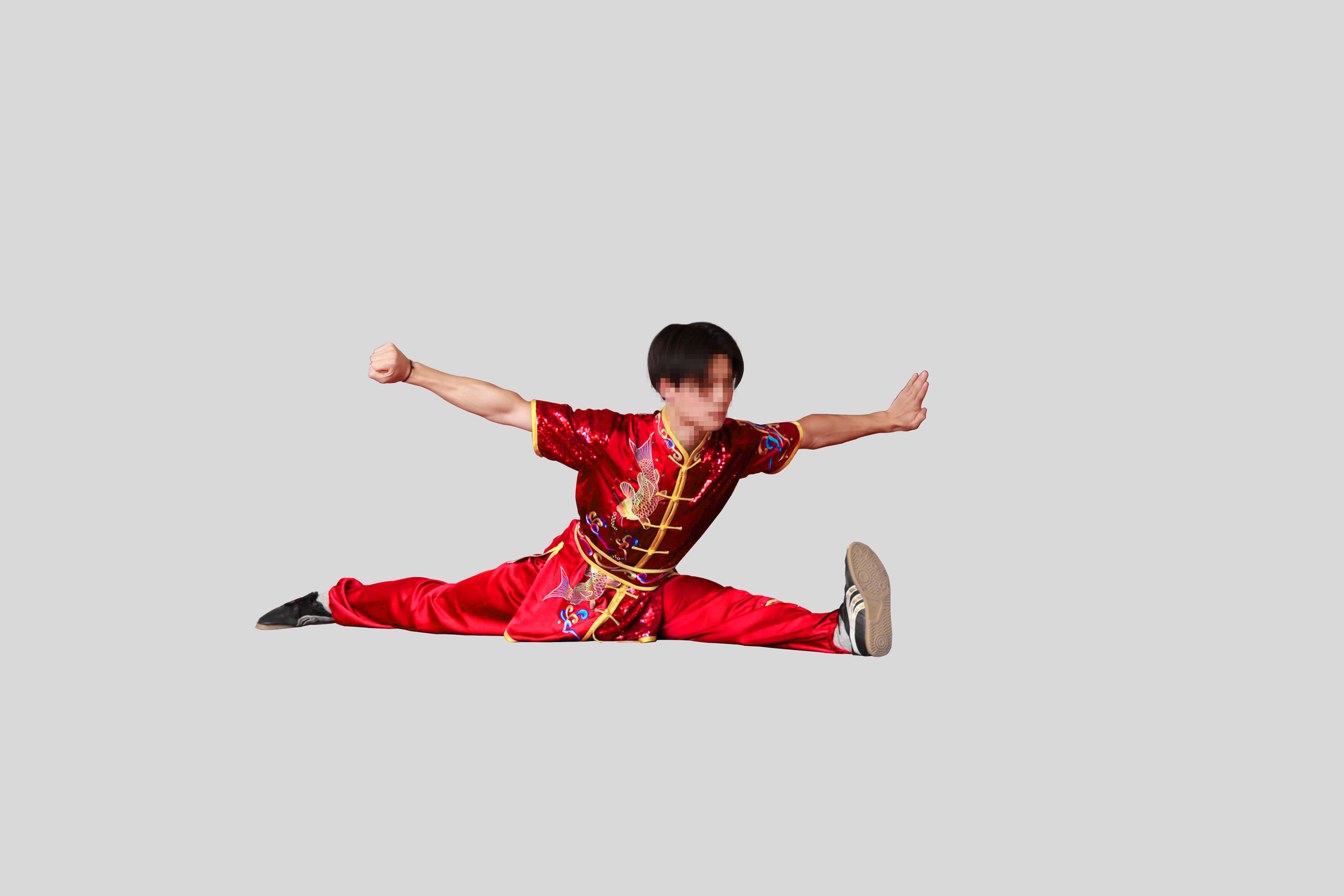

Supplement: S3 File — (ZIP) [file pone.0300893.s003.zip › athlete photos 3/longitudinal split(male in red).tif]

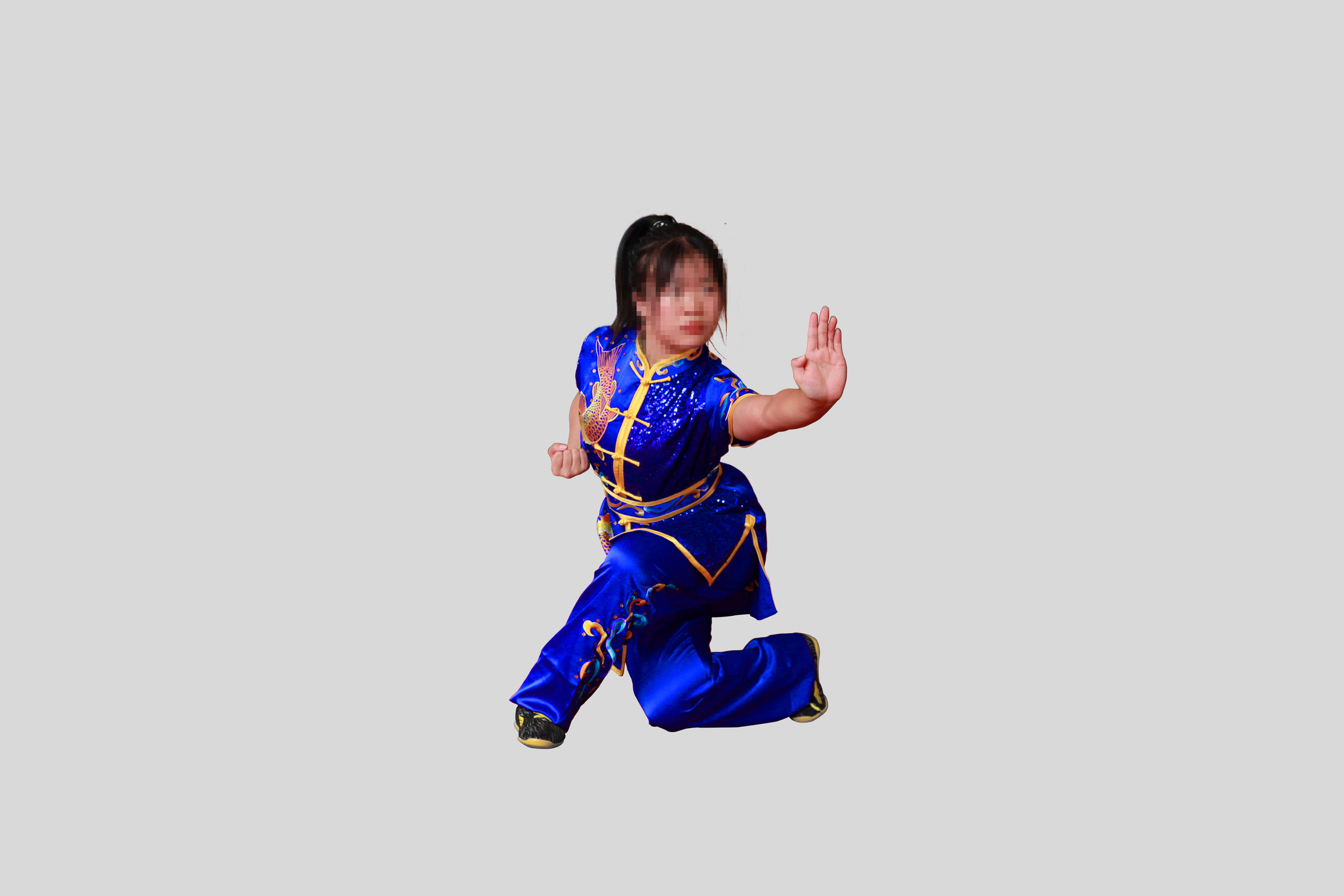

Supplement: S3 File — (ZIP) [file pone.0300893.s003.zip › athlete photos 3/rest-step push palm(female in blue).tif]

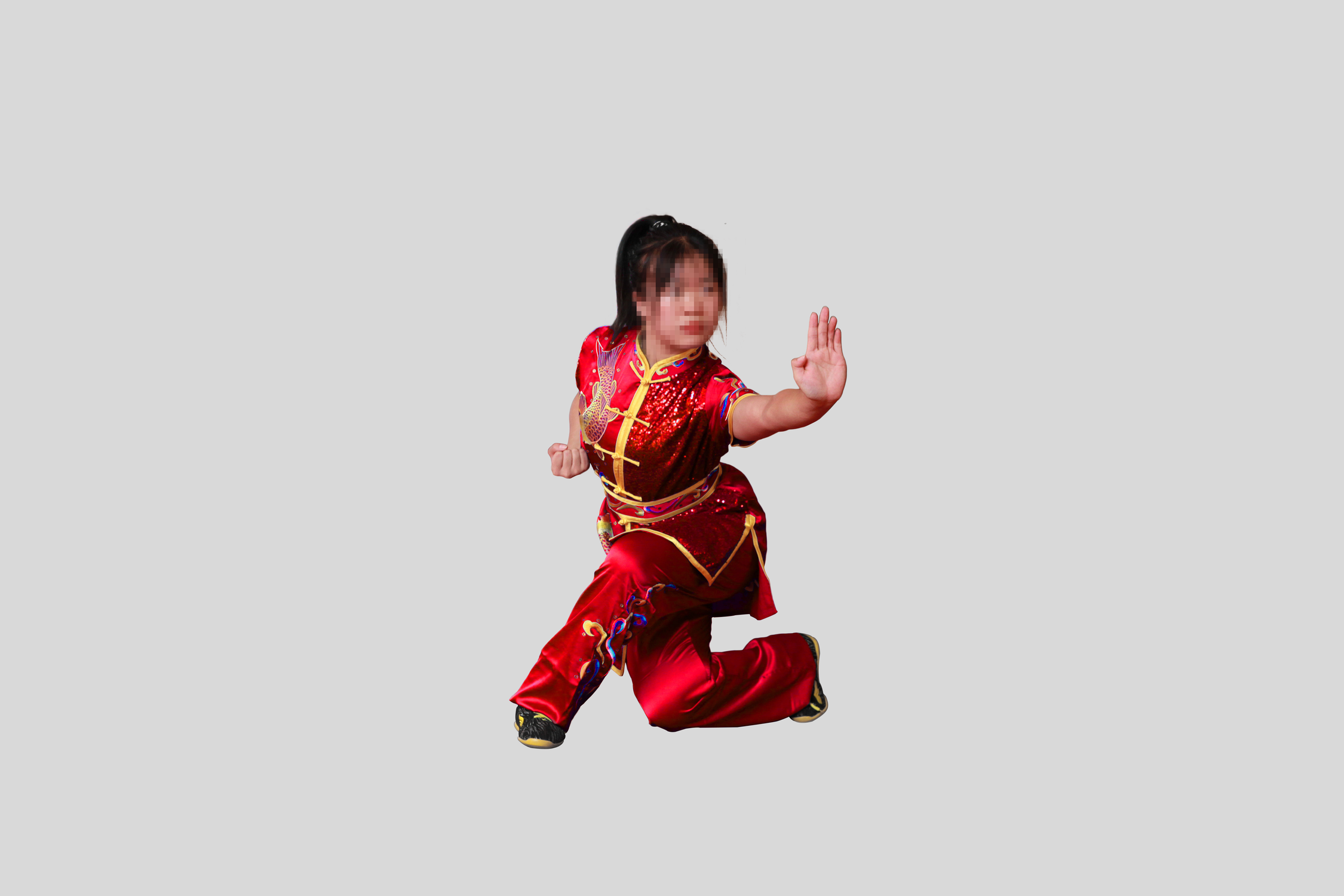

Supplement: S3 File — (ZIP) [file pone.0300893.s003.zip › athlete photos 3/rest-step push palm(female in red).tif]

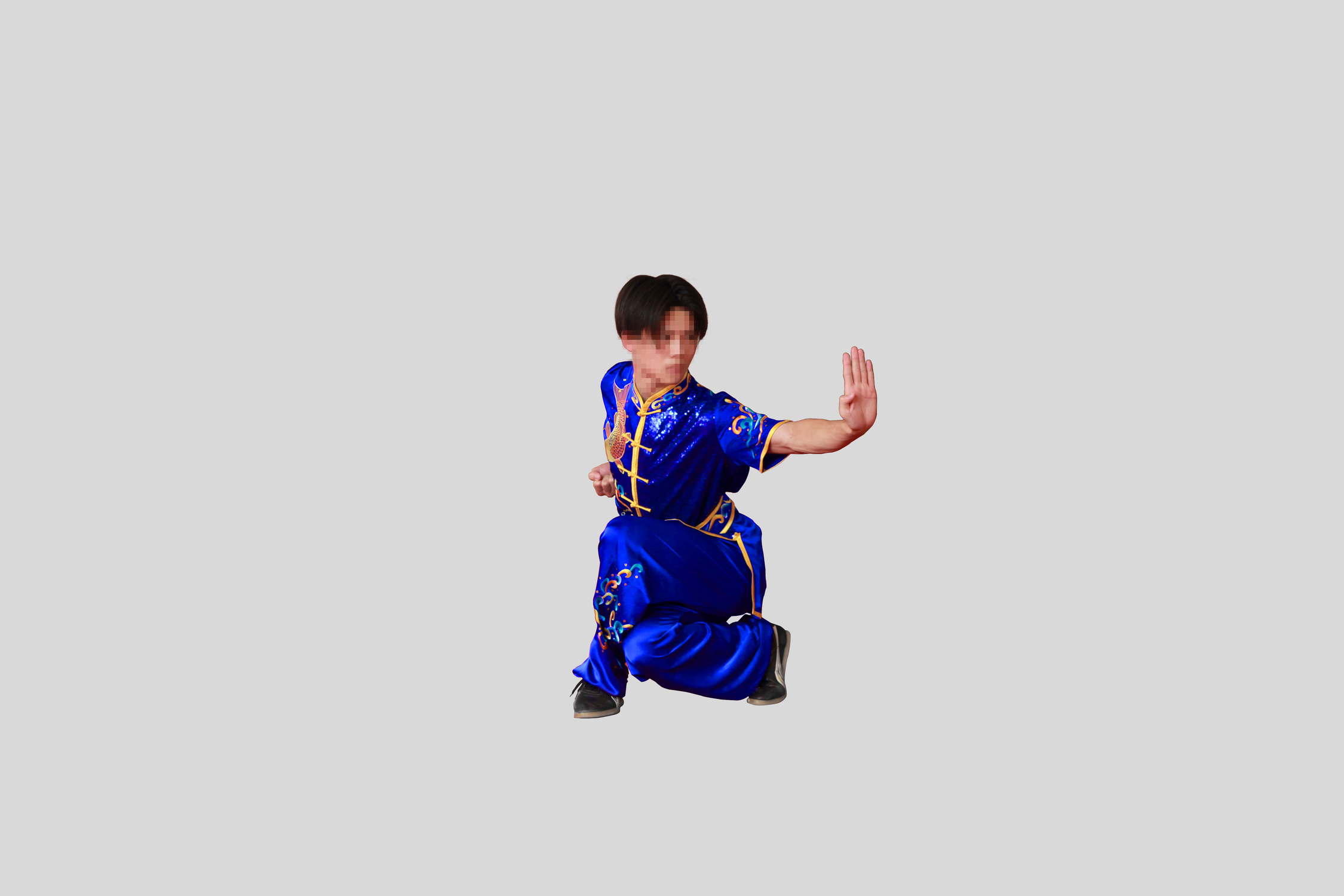

Supplement: S3 File — (ZIP) [file pone.0300893.s003.zip › athlete photos 3/rest-step push palm(male in blue).tif]

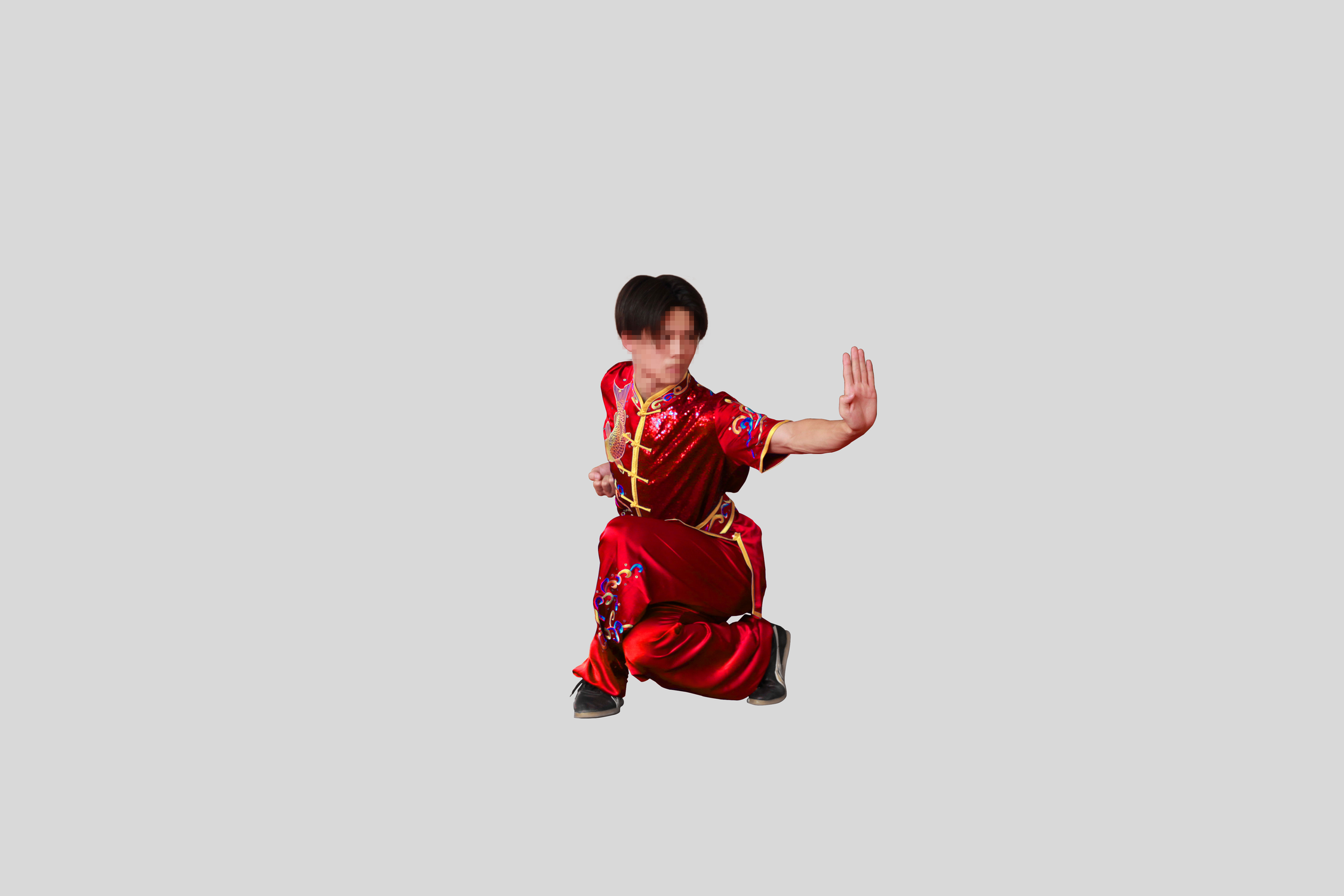

Supplement: S3 File — (ZIP) [file pone.0300893.s003.zip › athlete photos 3/rest-step push palm(male in red).tif]

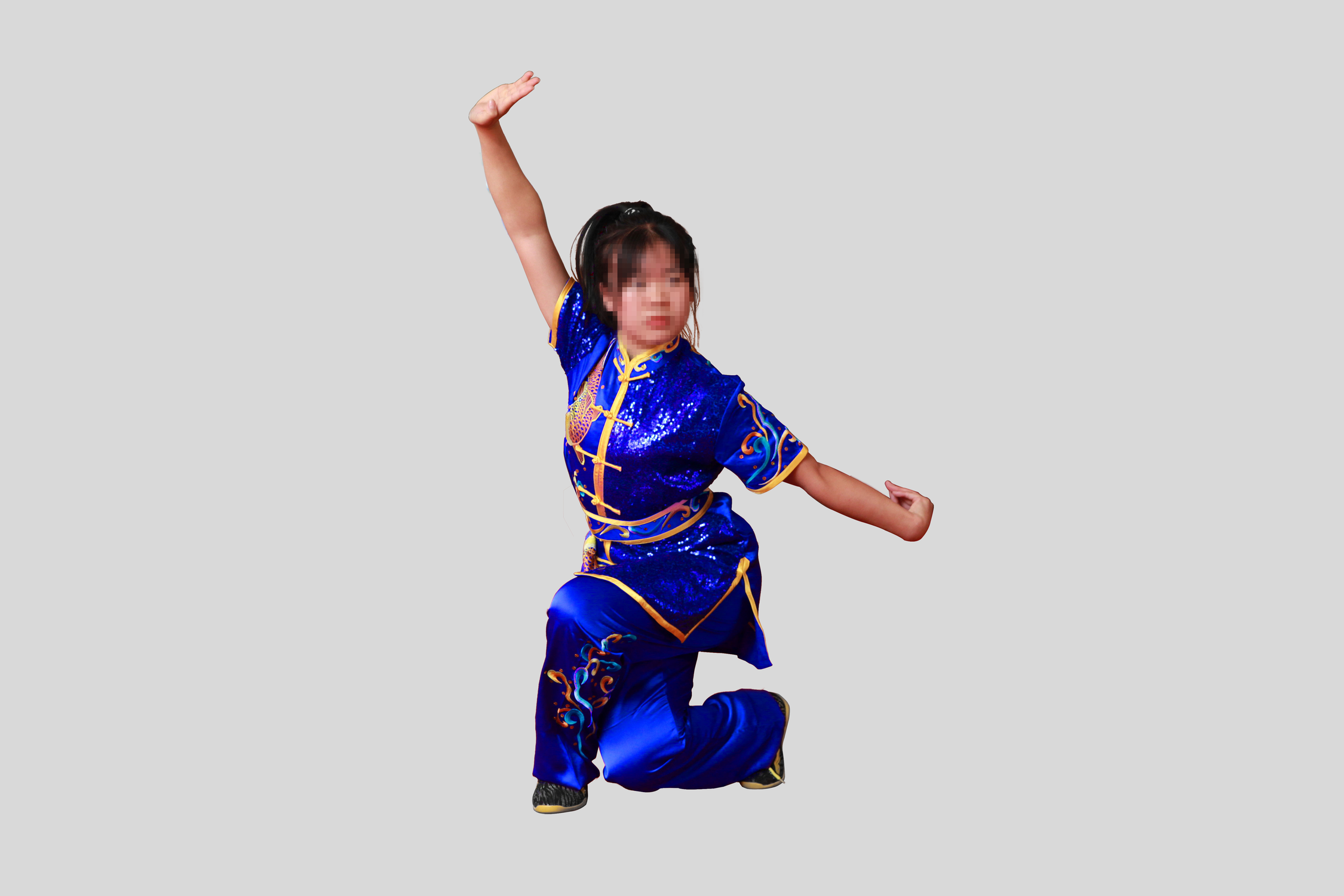

Supplement: S3 File — (ZIP) [file pone.0300893.s003.zip › athlete photos 3/rest-step show palm (female in blue).tif]

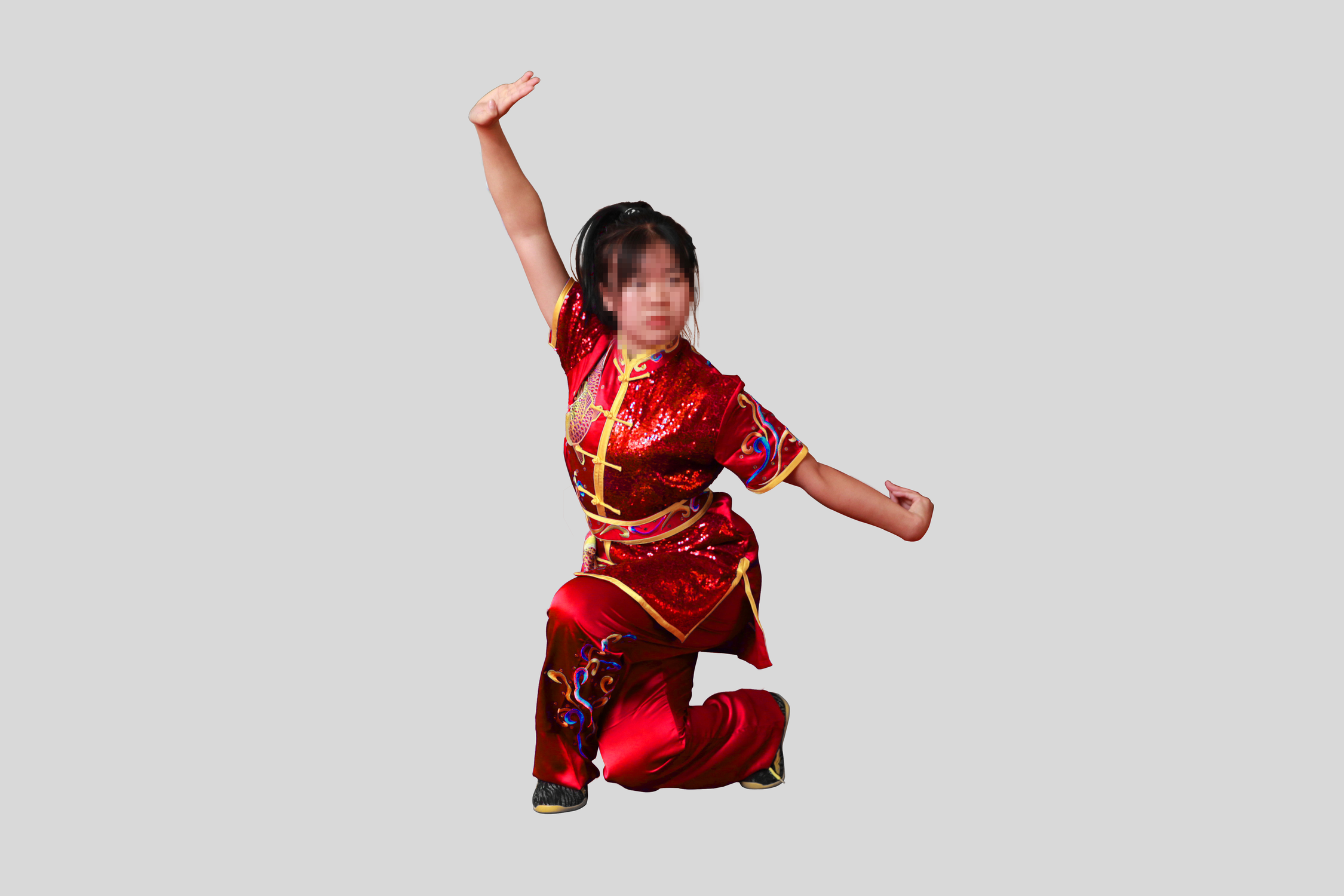

Supplement: S3 File — (ZIP) [file pone.0300893.s003.zip › athlete photos 3/rest-step show palm (female in red).tif]

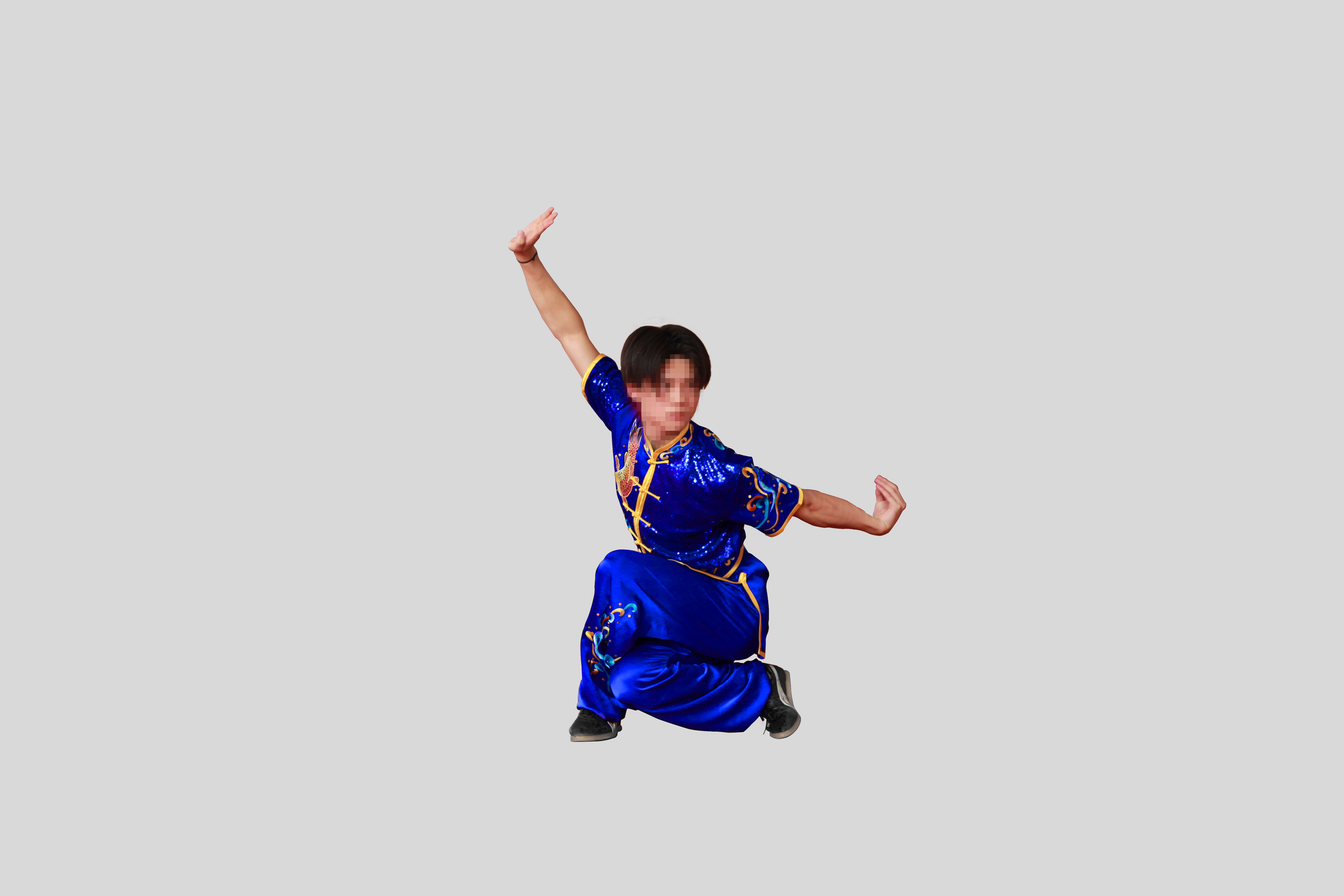

Supplement: S3 File — (ZIP) [file pone.0300893.s003.zip › athlete photos 3/rest-step show palm (male in blue).tif]

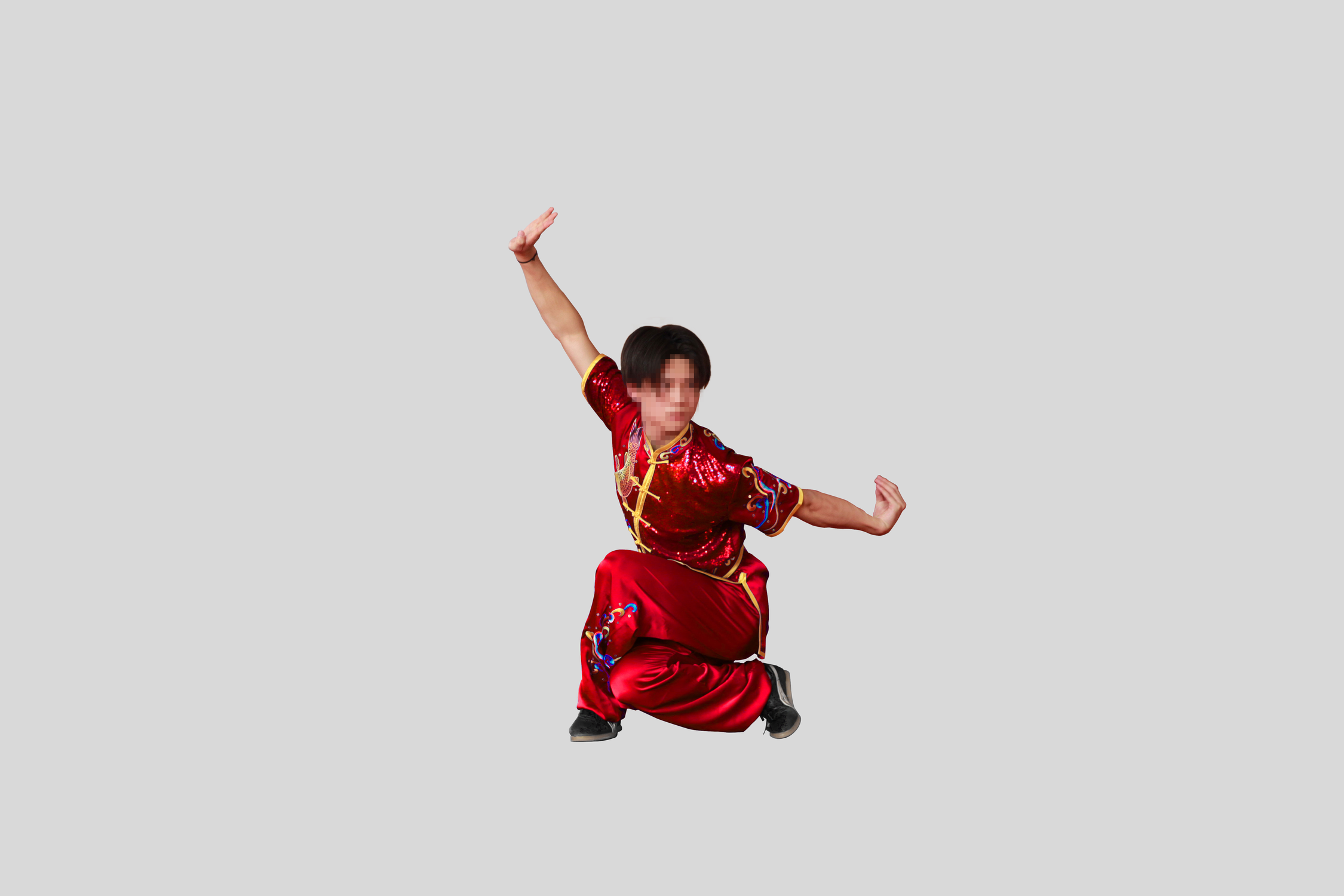

Supplement: S3 File — (ZIP) [file pone.0300893.s003.zip › athlete photos 3/rest-step show palm (male in red).tif]
