# Supplementary material for: Effect of the red uniform on the judgment of position or movement used in Wushu Routine, evaluated by practitioners of the modality
Source: PLoS One. 2024 Mar 21;19(3):e0300893. doi: 10.1371/journal.pone.0300893 (PMC10956778; doi:10.1371/journal.pone.0300893)
Supplement: Attachment — Submitted filename: Plos One Review.docx [file pone.0300893.s004.docx]

The study is interesting and provides relevant information for the study area, as well as for improving refereeing in Wushu, combat sports and other sports.

Some points should be considered, for example, the judging was carried out by Wushu practitioners and not by experienced referees. Another point, the analysis was carried out using photos of a position used in Wushu routines, but the analysis of performance judgment in competitions is much more complex.

Suggestion for the study:

**In the title:**

Instead of "sports performance", which is complex, it should be "Effect of the red uniform on the judgment of a position or movement used in Wushu routines, evaluated by practitioners of the modality."

**In the abstract:**

The authors describe that the study evaluates whether the color of the uniform can affect the evaluation of "referees", however the study evaluates whether the color of the uniform affects the evaluation of "wushu practitioners". It is suggested to adjust.

**In the introduction:**

It is suggested, at the end of introduction, before presenting hypotheses, to make the objectives of each of the experiments clearly.

**In the Method:**

Regarding the number of participants, was any type of sample calculation carried out? If it has not been done, add the information in the study limitations.

Would it be possible to include the participants' degree (level of knowledge) in Wushu, as well as the average time spent practicing in the modality?

The authors present just 4 photos, however, in the methods it is described that the participants evaluated 20 photos, it is suggested to place all the photos used in annex. It would be interesting to describe which positions or movements were evaluated.

Was any reproducibility assessment of the grades awarded carried out? Was a pilot study carried out to ensure the reproducibility of the assessments?

As in experiment 1, describe the degree (level of knowledge) and practice time in Wushu of the participants in study 2.

**In the discussion:**

In the discussion it is important to emphasize that sporting performance and referees' judgment is multifactorial, and the variable analyzed in the present study is one of these factors.

On page 21 of the study (Page 27 of the PDF), in line 466, where it says "imitations" insert "limitations".

**In conclusion:**

As in the title, the conclusion should describe that the effects of color (Red and blue) and the menstrual cycle (women in relation to ovulation vs. non-ovulation), are about judging movements or positions used in Wushu (Changquan), carried out by practitioners of the modality.

**In the figures:**

Improve the description of figures. Insert titles in the figures, indicate where significant differences were observed (e.g. *). The figures must be self-explanatory.
