# Supplementary material for: Effect of the red uniform on the judgment of position or movement used in Wushu Routine, evaluated by practitioners of the modality
Source: PLoS One. 2024 Mar 21;19(3):e0300893. doi: 10.1371/journal.pone.0300893 (PMC10956778; doi:10.1371/journal.pone.0300893)
Supplement: Attachment — Submitted filename: Response to Reviewers.docx [file pone.0300893.s006.docx]

We feel great thanks for your professional review work on our article. As you are concerned, there are several problems that need to be addressed. According to your nice suggestions, we have made extensive corrections to our previous draft, the detailed corrections are listed below.

Dear expert 1:

Thank you for your valuable suggestions on this article, in response to your comments, we have made the following changes.

1.Although the authors responded that they did not carry out a reproducibility analysis of the assessments and did not carry out a pilot study, I recommend that this information be included in the limitations of the study. These should even be recommendations for future studies.

Thank you for your advice. We have added the issue of no reproducibility analysis to the limitations section, and we think that future research could explore the reliability and reproducibility of this study with experienced referees as participants.

2. As was done with the database, I suggest also making the 20 images used in the study available for public access.

Thank you for your advice. We have uploaded the figures from the experiment to a public database (figshare), specifically “10.6084/m9.figshare.25284670".

Dear expert 2:

We sincerely appreciate the valuable suggestions you have given us! In response to your suggestions, we have made the appropriate changes.

1.Abstract:Please, on line 35 in the abstract, specify the number of study participants

Thank you for your advice. On line 35 in the abstract, we added the number of study participants.

2.In Tables 5 and 6 identify the y-axis label (Quality of the athlete's movements). I suggest redoing the titles of Figures 5 and 6, for example, in Figure 5 it could be: Assessment of the movements of male athletes considering the color of the athlete's uniform and the sex of the evaluator. In addition, you should place information about the p value below the Figures in Note format, not in the title.

Thank you for your advice. We reworked the figures and made changes to the titles and notes on the figures.
